# Microbial community profiling shows dysbiosis in the lesional skin of Vitiligo subjects

**DOI:** 10.1038/srep18761

**Published:** 2016-01-13

**Authors:** Parul Ganju, Sunil Nagpal, MH Mohammed, P Nishal Kumar, Rajesh Pandey, Vivek T Natarajan, Sharmila S. Mande, Rajesh S. Gokhale

**Affiliations:** 1 CSIR-Institute of Genomics and Integrative Biology (IGIB), Mathura Road, New Delhi, India; 2 National Institute of Immunology, Aruna Asaf Ali Marg, New Delhi, India; 3 Bio-Sciences R & D Division, TCS Innovation Labs, Tata Research Development & Design Centre, Tata Consultancy Services Ltd., 54-B Hadapsar Industrial Estate, Pune, India; 4 CSIR Ayurgenomics Unit-TRISUTRA, CSIR- Institute of Genomics and Integrative Biology (IGIB), Mathura Road, New Delhi, India; 5 Jawaharlal Nehru Centre for Advanced Scientific Research, Bangalore, India

## Abstract

Healthy human skin harbours a diverse array of microbes that comprise the skin microbiome. Commensal bacteria constitute an important component of resident microbiome and are intricately linked to skin health. Recent studies describe an association between altered skin microbial community and epidemiology of diseases, like psoriasis, atopic dermatitis etc. In this study, we compare the differences in bacterial community of lesional and non-lesional skin of vitiligo subjects. Our study reveals dysbiosis in the diversity of microbial community structure in lesional skin of vitiligo subjects. Although individual specific signature is dominant over the vitiligo-specific microbiota, a clear decrease in taxonomic richness and evenness can be noted in lesional patches. Investigation of community specific correlation networks reveals distinctive pattern of interactions between resident bacterial populations of the two sites (lesional and non-lesional). While Actinobacterial species constitute the central regulatory nodes (w.r.t. degree of interaction) in non-lesional skin, species belonging to Firmicutes dominate on lesional sites. We propose that the changes in taxonomic characteristics of vitiligo lesions, as revealed by our study, could play a crucial role in altering the maintenance and severity of disease. Future studies would elucidate mechanistic relevance of these microbial dynamics that can provide new avenues for therapeutic interventions.

Human skin surface provides favourable microenvironment for a myriad of microbial communities to flourish that constitute cutaneous microbiome^1^. Skin gets colonized by microbes at time of birth whose composition gets altered after subsequent exposure to the environment^2^. Host-microbe interactions have been shown to play a key role in human physiology, with commensal microbial population forming an essential component of biological processes like immunity and metabolism^3^,^4^. Characterization of cutaneous bacterial populations has revealed a remarkable difference in community composition based on body site sampled, suggesting that localised skin physiology plays a key role in dictating associated bacterial profiles^5^,^6^. The resident microbial community, in turn, influences localized responses to injury, infection and autoimmunity^7^. Besides having a symbiotic relationship with the tissue where they harbour, microbes are also believed to influence, in part, the course of associated disease. Increasingly various dermatological disorders like atopic dermatitis and psoriasis are being linked to changes in the structure of skin microbiome^8^,^9^,^10^.

Vitiligo is a chronic skin disorder that is characterized as patchy loss of skin pigmentation due to melanocyte death^11^,^12^. The inability of melanocytes to repigment lesional skin suggests localised active mechanisms preventing remigration of melanocytes. Exact cause of the disease remains an enigma as mechanisms underlying melanocyte disappearance are still not known. However, several hypotheses have been proposed to explain the pathogenesis of vitiligo^11^,^13^. Autoimmune attack of melanocytes is thought to be a major factor driving skin depigmentation, and existing treatment strategies are therefore focussed on ameliorating excessive immune activity. However, limited success of current treatment strategies poses a question on autoimmune mechanism of melanocyte loss. None of the current hypotheses can thus independently address the clinical and phenotypic progression of disease^14^. Vitiligo can be considered as a complex multifactorial syndrome where multiple disorders can result in the same phenotype, i.e. loss of melanocytes^15^.

Several studies have also associated alterations in skin physiology with loss of pigmentation in Vitiligo. For instance, lesional keratinocytes have been shown to possess altered growth and functional patterns under *in vitro* conditions^16^. Redox perturbations in the affected regions, with millimolar levels of hydrogen peroxide, have also been reported^17^,^18^. To understand the association of localized alterations in Vitiligo skin with cutaneous microbiota, we have explored the microbial community profiles of the lesional and non-lesional skin patches of vitiligo subjects using 16S rRNA phylotyping. Our analysis suggests dysbiosis to be associated with vitiligo lesions, with decreased diversity of cutaneous microbiota. Altered network topology is observed between the two microbial communities, indicating a drastic change in community dynamics. Our study is the first attempt to delineate “Vitiligo-specific microbiota” and provides a conceptual framework for understanding relevance of microbe interactions in diseased state that could play a crucial role in determining the pathophysiological state.

## Results

### Community composition of lesional and non-lesional skin of Vitiligo subjects

To assess core taxonomic characteristics of lesional and non-lesional skin, we have generated profiles of V1-V2 hypervariable region of 16s rRNA gene from 10 different vitiligo subjects (20 paired sites). Pre-processing of raw sequence data yielded a total of 869,583 high-quality sequences. RDP taxonomic analysis indicated presence of 21 distinct phyla. Rarefaction plot revealed curves that did level off but did not reach an ideal asymptote (Supplementary Figure S1). However, Good’s coverage of >97%, indicated reasonable sequencing coverage (Supplementary Table S1).

**Figure 1:**
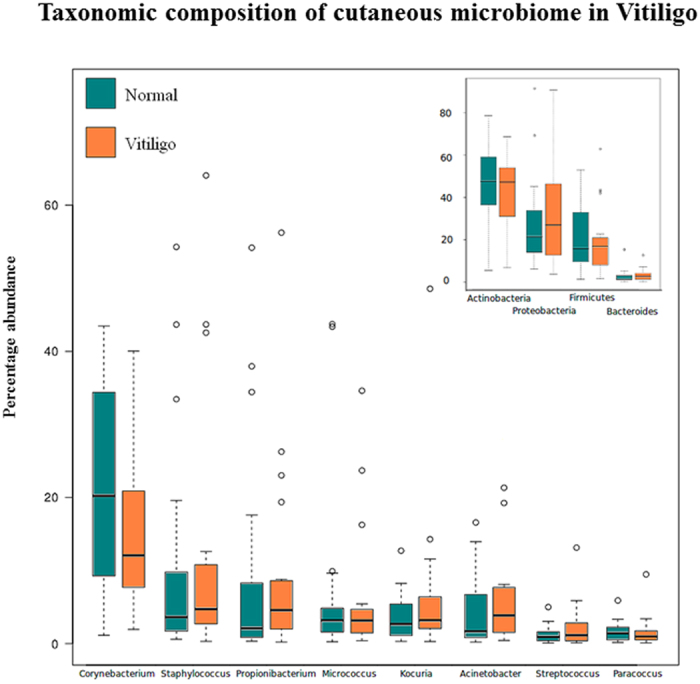
Taxonomic composition of cutaneous microbiome in Vitiligo. Boxplots representing relative abundance analysis of the bacterial taxa discovered in samples obtained from Non-Lesional and Lesional sites, at genus (main) and phylum (inset) levels. Taxa with minimum median abundance of 1% were used for the comparison.

The bacterial communities in both skin types are dominated by four phyla, with around 85% of sequences assigned to them. These include: Actinobacteria, Proteobacteria, Firmicutes and Bacteroidetes, and are consistent with previous observations of composition of healthy skin microbiota^6^ (Fig. 1, inset). The phylum Actinobacteria, with median abundance of greater than 45%, is observed as the most abundant phylum in both sample classes. Of the 702 unique microbial genera with varying abundance proportions, 173 in non-lesional and 160 in lesional skin are observed to have non-zero median abundance. Greater than one-third of taxonomic assignments belong to eight major genera: Corynebacterium, Staphylococcus, Propionibacterium, Micrococcus, Kocuria, Acinetobacter, Streptococcus and Paracoccus (Fig. 1).

**Figure 2:**
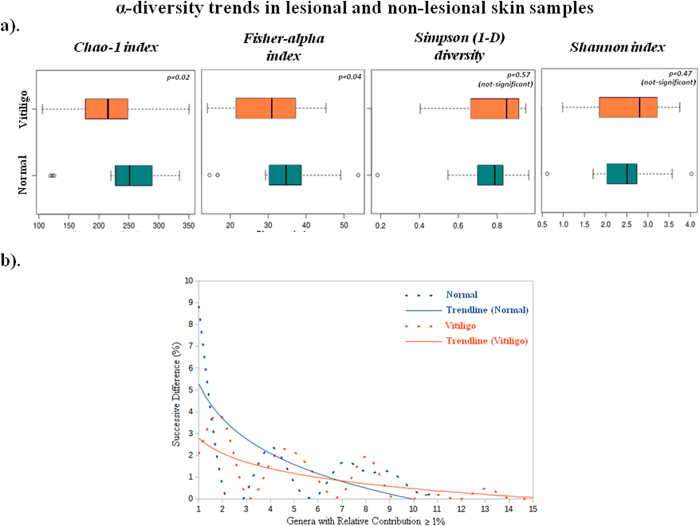
α-diversity trends in lesional and non-lesional skin samples. (**a**) Boxplots illustrating the comparison of diversity indices (Chao-1, Fisher, Simpson1-D and Shannon index) between Non-Lesional and Lesional samples. (**b**) Comparison of differences between successive relative contribution values (of the ordered genera) in Non-Lesional and Lesional samples. Only those genera were considered for calculating successive differences in relative contributions that had a minimum relative contribution of 1%.

Alpha diversity metrices for richness (Chao-1, Fisher), evenness (Simpson) and diversity (Shannon) were calculated for two skin types (Fig. 2a). While differences in Shannon and Simpson indices are statistically insignificant, average Chao-1 and Fisher indices indicate higher (p <0.1) species richness in non-lesional samples as compared to their lesional counterparts. The evenness pattern was further analysed by comparing ‘relative contribution’ values as a metric^19^. Genera having atleast 1% relative contribution in both lesional and non-lesional samples were identified and sorted (in a descending order) in terms of their relative contributions. Differences between successive relative contribution values were then computed and plotted. Slope of the curve obtained by plotting these successive differences would serve as an indicator of any abundance skew in the population, with a steep slope indicative of presence of one or more dominant genera in the population. Logarithmic trend lines for the two skin types clearly indicate presence of a few dominating genera in non-lesional samples (Fig. 2b). In contrast, lesional skin possesses a fairly even distribution of various genera. To validate the above observations, the number of taxa having at least 0.01%, 0.1%, and 1% relative contributions were calculated in individual sample classes (Supplementary Table S2). A sample class having an even distribution of taxa would have a higher number (and percentage) of taxa. In line with the previously indicated trend, results of this analysis also indicate a higher number of taxa in lesional samples at all thresholds.

### Core microbiota comparison between lesional and non-lesional skin of vitiligo subjects

A core skin microbiome of OTUs and RDP abundance data for both skin types was identified. RDP abundance data indicate presence of 23 core genera in non-lesional skin as opposed to 19 in lesional, with 18 genera in common (Supplementary Figure S2a). While Methylobacterium constitutes the core genus exclusive to lesional skin, Anaerococcus, Microbacterium, Streptophyta and Nocardiodes constitute core genera exclusive to non-lesional samples. Similar analysis performed using OTU abundance data indicate 14 OTUs as common to both skin types (Supplementary Figure S2b). Two OTUs belonging to genera Janibacter and Brevundimonas were however observed to be exclusive to non-lesional samples. The OTUs belonging to Enhydrobacter, Paracoccus and Staphylococcus constitute the core genera exclusive to lesional samples.

### Vitiligo-specific microbiota analysis

To stratify lesion specific microbiota, we identified (RDP-derived) microbial taxa having significantly different abundances as mentioned in the methods section. Overall, 39 taxa (21 genera, 9 families, 5 orders and 4 classes) are observed to have a statistically significant difference in their relative abundance between the two skin types (Table 1). Except for Corynebacterium, all significantly different bacterial genera are observed to have median percentage abundance less than 1%. Amongst the significantly differentiating taxa with a median (%) abundance > =1, Corynebacterium (genus) and Corynebacteriaceae (family) are observed to have higher abundance in non-lesional samples, whereas Flavobacteriales (order), Gammaproteobacteria (class) and Flavobacteria (class) have significantly higher abundance in lesional samples.

Similar analysis on OTU level abundance data resulted in identification of 27 significantly differentiating OTUs (Table 2). The OTU corresponding to Propionibacterium (OTU_32), constituting a major proportion of bacterial abundance in both sample classes (>5%), is a significantly differentiating OTU. Interestingly, significance tests on RDP data did not identify Propionibacterium as a significantly differentiating taxon. This observation may be attributed to the resolution of sequences belonging to Propionibacterium into multiple OTUs, signifying multiple species/strains under this genus. Only 9 significantly differentiating OTUs have median (%) abundance greater than 0.1%, suggesting that the key differences between the lesional and non-lesional samples are contributed by sparsely abundant bacteria.

**Figure 3:**
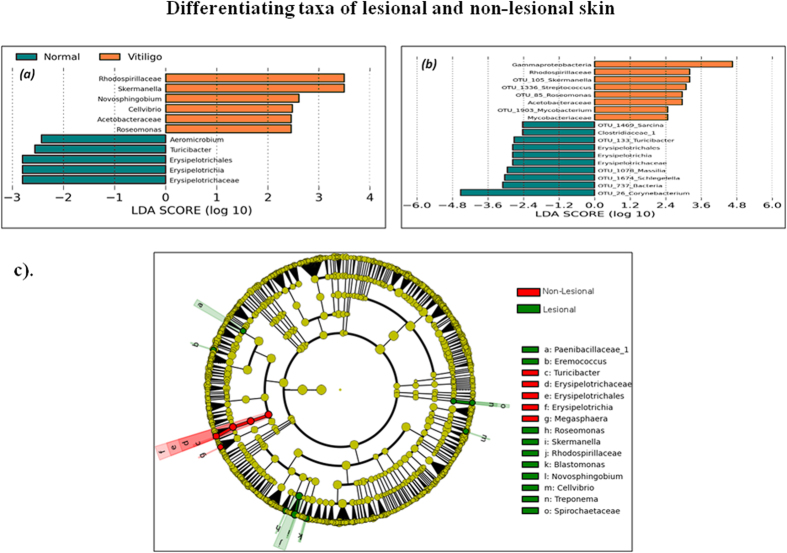
Differentiating taxa of lesional and non-lesional skin. Comparison of LDA effect size of the significantly differentiating microbial taxa (**a**) RDP level and (**b**) OTU level between Non-Lesional and Lesional samples. LefSe package was used to generate the LDA effect size with LDA cut-off =2. Wilcoxon p value cut-off of 0.1 was used for differentiating feature analysis through LefSe. (**c**) Cladogram illustrating the phylogenetic relationship amongst the significantly differentiating microbial taxa (RDP level) between Non-Lesional and Lesional samples, deduced using LefSe package.

Differentiating features between the two skin types were also identified using Linear Discriminant Analysis (LDA)-based LefSe approach^20^. Results of this approach (using RDP abundance data) indicate 11 differentiating features at a p-value cut-off of less than or equal to 0.1 for Kruskal-Wallis, and pairwise Wilcoxon test between lesional and non-lesional samples with an absolute LDA score >2. The differentiating taxa obtained were observed to share a noticeable consensus with results obtained through independently performed Wilcoxon signed rank test (Fig. 3a). Seven taxa, namely, Skermanella, Novosphingobium, Roseomonas, Cellvibrio, Turicibacter, Erysipelotrichaceae and Erysipelotrichales are the common set of differentiating taxa. Given that final graphical representation of significantly differentiating taxa identified using LefSe’s LDA approach is based on comparison of the mean abundance values, taxon Skermenalla is depicted as having higher abundance in lesional samples. In contrast, results depicted in Table 1, indicate an opposite trend. This is due to the use of median (%) abundance as the criterion for comparison. LefSe approach was utilized for differential taxa analysis using OTU level abundance data as well. Results of the analysis are illustrated in Fig. 3b indicating differentially abundant taxa with an absolute LDA score > 2. Fig. 3c, represents a cladogram highlighting the lineage of the deduced differentiating features.

### Ordination Analysis of cutaneous microbiome of lesional and non-lesional skin

To identify patterns of variation across the two skin types, ordination analysis was performed. Clustering of samples is observed according to subjects, rather than disease status, suggesting individual-specific microbiome signatures (Supplementary Figure S3a). In 7 out of 10 subjects, greater than 75% of samples belonging to individual subjects are observed to belong to one cluster. Interestingly, the four major clusters (1, 2, 3 and 5) are also observed to contain a majority of samples from similar body locations i.e. Cluster 1: Waist; Cluster 2: Hands; Cluster 3 & 5: Legs (Supplementary Figure S3b). However, there appears to be a lack of pattern as per disease status of samples.

### Comparison between lesional and non-lesional bacterial composition using Intra-community network analysis

To evaluate correlation between individual members of the microbial community and assess associations between them, we performed intra-community network analysis. The resulting networks were analysed for various network properties (nodes, edges, density, diameter etc.,) and centrality measures (degree and betweenness) (Supplementary Table S3). Our results show that the correlation network generated using abundance data of non-lesional skin is enriched both in terms of number of nodes as well as edges indicating greater diversity as well as higher number of interactions between various members of the community (Supplementary Figure S4a,b). Interestingly, maximum degree in the network of non-lesional samples is observed to be significantly higher than that obtained with lesional samples. The common set of interactions (core-interactions) is observed to be sparse, indicating a drastic change in the community dynamics.

**Figure 4:**
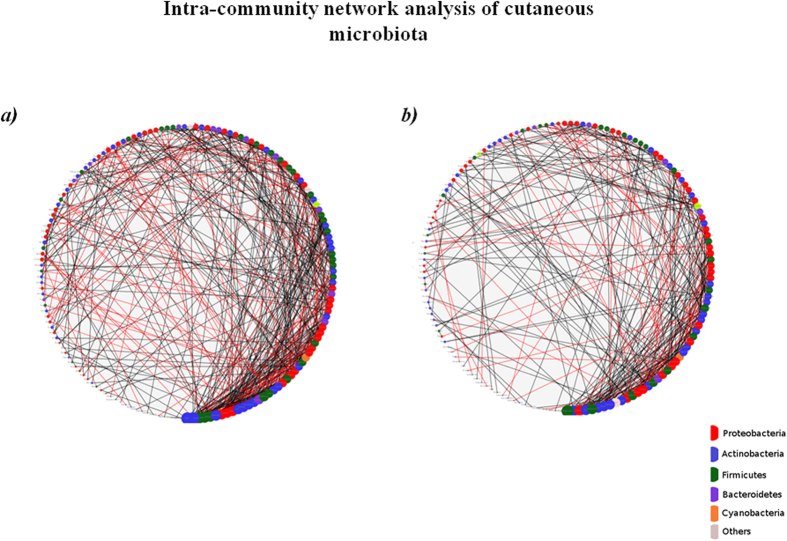
Intra-community network analysis of cutaneous microbiota. Degree sorted circular layout of the networks generated for (**a**) Non-Lesional and (**b**) Lesional sample sets. Nodes are sorted according to their degree in such a way that the size of the node serves as an index of the magnitude of its degree. Color of the nodes represents their phylum affiliation.

We further analysed if nodes with higher degree (in either network) share any correlation with respect to their phylogenetic affiliation and phenotypic features like Gram nature, Sporulation, Oxygen requirement and Cell shape. For this purpose, a ‘degree threshold’ was defined in the following manner. Nodes having a degree that is at-least N% of the highest degree node were considered. The value of N was varied between 60–90% (Supplementary Table S4a,b). Figure 4a,b indicate circular layouts of both the networks wherein nodes are sorted and sized based on their degree (larger size for higher degree nodes). While taxa belonging to Actinobacterial lineages predominate the network of non-lesional skin, Firmicutes forms the dominant lineage of high degree nodes in lesional samples. Also, the contribution of taxa belonging to Proteobacterial lineages towards high degree nodes is higher in non-lesional sites as compared to lesional sites. These results maintain a consistent pattern at various degree thresholds. Interestingly, within the phylum Proteobacteria, while the majority of high degree nodes in non-lesional samples belong to class alpha-proteobacteria, the lesional samples were observed to be dominated by beta-proteobacteria and gamma-proteobacteria. Analysis at various phenotypic levels did not indicate any marked differences between pair of sample environments.

## Discussion

Skin forms an important ecosystem by providing conditions for microbial communities to flourish. The structure of cutaneous microbiome is dictated by localized topography of skin and is generally observed to remain relatively stable over time. Symbiotic interactions between the complex microflora and skin shape and modulate the innate immune response of the host^21^. Maintenance of the right kind of microflora is thus essential for healthy skin. Several studies have indeed catalogued modulation of cuataneous microbiome with allergic and inflammatory skin diseases, like atopic dermatitis and psoriasis^8^,^9^. It is therefore tempting to speculate that altered interaction between microbiota and host could play a critical role in deciding disease outcome.

The depigmenting disorder Vitiligo is characterized by loss of epidermal melanocytes in patches. This is coupled with high oxidative stress conditions in the lesional site that alters local skin physiology. Though the clinically unaffected skin appears normal, unpredictable spread of depigmentation indicates substantial instability of peripheral skin^22^,^23^. Further, it remains unclear if the modified skin biology and localized immune modulation in lesional patches has any association with the resident microflora. In the current study, we present the first comprehensive analysis of cutaneous microbial community profiles of vitiligo subjects. We observe a trend of decreasing diversity from non-lesional to lesional sites, with not much difference in evenness. This trend is conserved in lesional and non-lesional samples, irrespective of their anatomical environments. We have also identified disease-specific microbial taxa by comparing abundance profiles. We find a positive association of 13 specific OTU’s and inverse correlation of 12 OTU’s with lesional patches. Interestingly, most of the differentiating OTU’s belong to the low abundance taxa. Further, our ordination analysis suggests that individual-specific skin microbiome signatures predominate over disease specific changes, suggesting that the global microbial landscape remains unaltered in lesional skin and there appears to be a lack of pattern as per the disease status.

Although our initial analysis suggests that the association of microbial taxa with lesional skin in vitiligo is not robust, we have performed network analysis to obtain a heuristic picture of microbial diversity of non-lesional and lesional skin samples from vitiligo subjects. Surprisingly, the microbial communities associated with non-lesional and lesional skin show different network profiles, with higher number of interactions (as indicated by more number of edges) between the different members of bacterial community of non-lesional skin as compared to lesional skin sites. Interestingly, the microbial interaction network of lesional sites is completely different from that of non-lesional sites, which again signifies altered community dynamics at the lesional site. Our correlation network approach has revealed that microbes belonging to Actinobacterial lineages that have the highest degree of interactions in the non-lesional skin network are replaced by microbes belonging to phylum Firmicutes in the network generated using samples from lesional skin sites. Our network analysis thus suggests strong niche-specific co-occurrence patterns with specific hub organisms associated with non-lesional and lesional skin sites.

Topological differences in skin sites can give rise to microbial changes^5^,^6^. We have taken into account this criterion for most of the samplings. However, under rare circumstances where lesional patch was bigger in size, we have obtained samples from different sites. To ensure that site-specific variations do not contribute to the above results, we also performed detailed analysis on paired sites with similar anatomical environment (excluding *X1N1*, *X1N4*, *Y1N1*, *C1N1*, *Z1N1* pairs) (Supplementary Figure S5). Our results (Supplementary Figures S7 to S13 and Supplementary Tables 8 and 9) suggest that the microbial abundances observed in the subset of samples (15 pairs) from anatomically similar skin sites are similar to that observed during analysis of the whole sample set (20 paired samples).

In conclusion, our studies indicate differences in microbial community dynamics of the lesional and non-lesional sites of Vitiligo subjects, with greater diversity and higher association between microbial communities of the unaffected site. A key aspect of our study is analysis between matched sites from the same individual. This eliminates likelihood of variations arising from interpersonal differences and adds robustness to the analysis. However, it remains unclear if modulated microbiome reflects a cause or effect of altered skin physiology of the depigmented patches. This is the first study of microbial analysis from vitiliginous subjects and provides insights into the alterations and adaptations of bacterial networks in diseased state. This study is expected to aid in understanding the potential role of microbial community in vitiligo pathophysiology and is of potential therapeutic and diagnostic significance.

## Materials and Methods

### Subject recruitment and selection criteria

The study protocol was approved by Human ethics review committee of CSIR-Institute of Genomics and Integrated Biology. The procedures were carried out in accordance with the approved institutional ethics guidelines and are in agreement with Declaration of Helsinki principles. We obtained written consent from 10 patients with Vitiligo, all of who had not been exposed to antibiotics atleast 6 months or received treatment for Vitiligo for atleast 6 months prior to skin sample collection. Subjects ranged in age from 20 to 45 years and were not suffering from any apparent infection. Skin preparation included using only Dove soap for hygiene for 7 days, refraining from use of topical lotions/antiseptics/antibacterial soaps for 7 days, and avoiding any form of washing 24 hours prior to sampling.

During subject selection, the following inclusion criteria were considered

1. Moderate to widespread vitiligo in subjects.
2. Subjects willing to provide written consent for sample collection.
3. Male or female subjects - 18 years and above (not above 55 years at the time of study).

Exclusion criteria included:

1. Presence of active infection at the time of sample collection.
2. Suffering from autoimmune diseases like, rheumatoid arthritis or diabetes mellitus.
3. Undergoing Vitiligo treatment, like phototherapy, corticosteroids (topical or oral) etc. at least 6 months prior to sample collection.
4. Use of topical antibiotics or topical steroids.
5. Use of the following drugs at least 6 months prior to sampling.
  - Systemic antibiotics (Oral, intravenous, intramuscular)
  - Oral corticosteroids
  - Cytokines or immunosuppressive agents

Sample nomenclature was as follow: Subject ID-involved versus uninvolved site-site number. For example, A1N1 is sample obtained from non-lesional skin of subject A1 at site 1. Corresponding sample from lesional skin would be A1V1. Supplementary Figure S5 illustrates sampling locations along with corresponding identifier tags. Supplementary Figure S5 summarizes subject information recruited in the study.

### Genomic DNA extraction and 16S rRNA gene pyrosequencing

Genomic DNA was extracted from all swabs using PowerSoil DNA Isolation kit (MoBio, Carlsbad, CA). For each sample, V1-V2 region of the 16S rRNA gene was amplified using modified primer set described earlier^24^,^25^.The forward primer (5′GCCTTGCCAGCCCGCTCAGTCAGNNNNNNNNNNNAGTTTGATCCTGGCTCAG-3′) contained the 454 Life Sciences primer B sequence, a four base linker sequence (TCAG) and the broadly conserved bacterial primer 27F. A unique 11-nucleotide Molecular Identifier (MID) barcode used to tag each PCR product was added for multiplexing during sequencing reaction. MID sequences were from Roche (MID 1 to 12) and IDT (MID 13 to 20). The reverse primer (5′-CCTATCCCCTGTGTGCCTTGGCAGTCTCAG TGCTGCCTCCCGTAGGAGT -3′) contained the 454 Life Sciences primer A sequence, the broad-range bacterial primer 338R, and a ‘TCAG’ linker sequence inserted between primer A and the rRNA primer. As negative control, we isolated genomic DNA from swabs dipped in lysis buffer using the protocol mentioned in the manuscript. We then amplified V1-V2 region using standard PCR reactions. However, we could not get any amplification in these samples, suggesting no background amplification from the swab/lysis buffer. This was a prerequisite quality control step undertaken to ensure removal of false positives. Thermal cycling consisted of initial denaturation at 94 °C for 5 minutes followed by 35 cycles of denaturation at 94 °C for 30 seconds, annealing at 59 °C for 50 seconds, and extension at 72 °C for 45 seconds, with a final extension of 10 minutes at 72 °C. Purified amplicons were combined in equimolar ratios and emulsion PCR followed by pyrosequencing was carried out using primer A and reagents for 454 GS FLX on a 454 Life Sciences Genome Sequencer FLX Plus instrument (Roche) according to manufacturer’s instructions. Supplementary Tables S6,S7 represents the tabulated summary of MID sequences used for primer designing and primers used for each sample, respectively.

### Pre-processing of sequencing data

In-house scripts (designed at TCS Innovation Labs) were employed for segregating and filtering sequence data. The following parameters were used during this process: exact match to respective barcodes during segregation, minimum (mean) phred score of 20 and a minimum sequence length of 100bp during quality filtering of fastq records. The resulting fasta files were then provided as input to the V-Xtractor 2.0 program for retaining the V1-V2 specific region in each sequence^26^.

### Taxonomic profiling and OTU clustering

Taxonomic classification of sequences in each sample was performed using the Ribosomal Database Project (RDP) classifier (version 2.2); bootstrap threshold of 80%. OTU clustering was performed using CROP^27^. Taxonomic assignment of individual OTU clusters was performed using the procedure illustrated in Supplementary Figure S6. Taxonomic and OTU abundance data obtained were used for all analyses discussed in the subsequent sections.

### Core Taxa Analysis

All microbial genera which were consistently represented at a minimum abundance of 0.1% in at least 80% of samples, were affiliated as core taxa.

### Differential taxa analysis using bootstrapped approach

Taxa/OTU’s with significant different relative abundance between lesional and non-lesional skin were analysed using a bootstrapped approach as described below.

**Step 1:** 50% of the samples were randomly chosen from each class (i.e. lesional and non-lesional) of datasets.

**Step 2:** In each class, the median abundances of constituent taxa/OTU in the randomly chosen subset of samples were computed.

The above steps were repeated 40 times to obtain 40 values of median abundances for each taxon in each class. Wilcoxon rank-sum test was then carried out between the obtained median abundance values (for each taxon) in both classes and taxa with significantly different abundance were identified using Benjamini-Hochberg (BH) p-value correction at an FDR of 0.0001.

The entire procedure described above was bootstrapped 1000 times. Taxa which were observed as having a significantly different abundance (post BH correction) in at least 99.5% of iterations were retained.

### Network Analysis

Rank normalized RDP taxa-abundance data was used for plotting networks between identified genera in microbial community of each sample class. Networks were generated based on the pair-wise correlations between rank normalized abundances of various members constituting the microbial community. Positive and negative interactions between all pairs of genera in a particular class of samples (lesional or non-lesional) were obtained using Spearman’s correlation coefficient as an index. Since ties were observed in the computed ranks, Spearman’s correlation coefficient was calculated using the following mathematical relationship (Eq. 1):


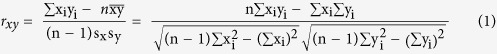


where,

x = 1^st^ taxon;

y = 2^nd^ taxon;

r_xy_ = Spearman’s correlation coefficient between taxon x and taxon y;

x_i_ = Normalized Rank of x in ith sample;

y_i_ = Normalized Rank of y in ith sample;

s = Standard deviation;


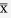
 = Mean rank of x across all samples of a particular class;


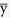
 = Mean ank of y across all samples of a particular class; and

n = Number of Samples.

Positive and negative interactions between a pair of taxa were tagged according to the critical r-value obtained at 99% confidence level. All correlation values (r) between the critical r-value and 1 were treated as positive correlations, and those between −1 and negative of the critical r-value were treated as negative correlations. Values between critical r-value and negative critical r-value were treated as insignificant correlations, and didn’t contribute towards creation of edges in the network. Correlations were plotted in Cytoscape 3.0.2 to visualize and study intra-community network characteristics^28^. Each network was analyzed for various phenotypic properties like Gram Nature, Sporulation, Cell Shape, Oxygen Requirement etc. Phylogenetic affiliations of microbes corresponding to nodes in the generated networks were also obtained and analyzed.

### Multivariate Statistical Analyses

The RDP and OTU abundance data, obtained for lesional and non-lesional samples, were analyzed for identifying taxa and OTUs having significantly different abundances (Wilcoxon signed rank test; p< = 0.1). Furthermore, Linear Discriminant Analysis (LDA) approach implemented within the LefSe tool was also employed for identifying significantly differentiating features (i.e. taxa/OTU’s) between the two sample classes^20^.

## Additional Information

**How to cite this article**: Ganju, P. *et al.* Microbial community profiling shows dysbiosis in the lesional skin of Vitiligo subjects. *Sci. Rep.* **6**, 18761; doi: 10.1038/srep18761 (2016).

## Supplementary Material

Supplementary Information

## Figures and Tables

**Table 1:**
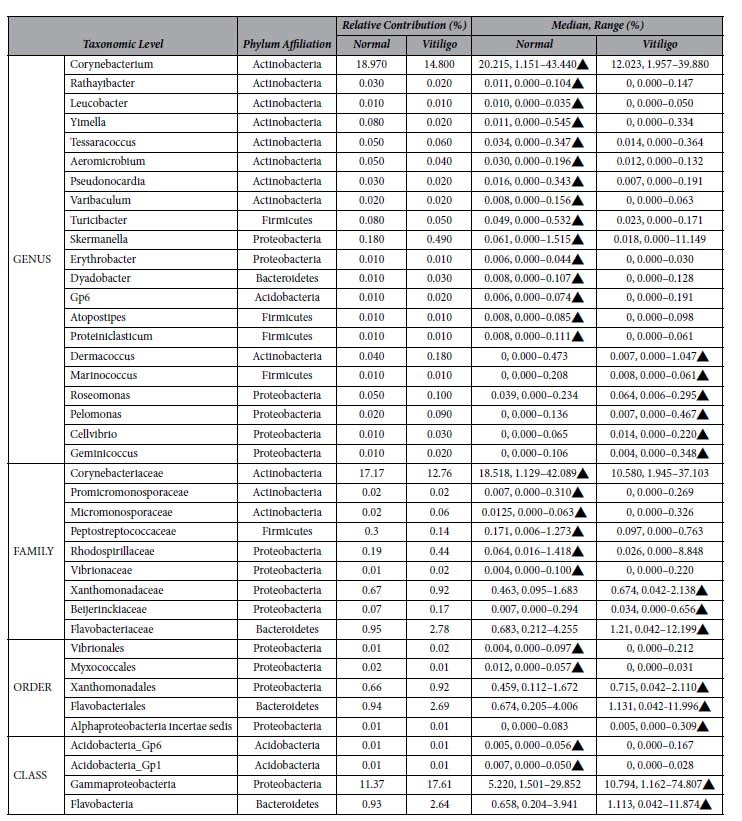
Differentially abundant taxa (RDP classification) between lesional and non-lesional skin.

A list of differentially abundant taxa (RDP classification) between lesional and non-lesional skin obtained using the Wilcoxon test coupled to a bootstrapping approach. In each iteration of the bootstrap method, taxa with significantly different abundance were initially identified using Benjamini-Hochberg p-value correction at an FDR of 0.0001. Subsequently, taxa which were observed as having a significantly different abundance (post BH correction) in at least 99.5% of iterations were retained.

**Table 2:** Differentially abundant OTU’s between lesional and non-lesional skin.

*OTU*	*Phylum Affiliation*	*Relative Contribution (%)*		*Median, Range (%)*	
		*Normal*	*Vitiligo*	*Normal*	*Vitiligo*
OTU_105_Skermanella	Proteobacteria	0.160	0.400	0.056, 0.000–1.086▲	0.014, 0.000–7.974
OTU_128_Jeotgalicoccus	Firmicutes	0.050	0.020	0.007, 0.000–0.340▲	0.000, 0.000–0.312
OTU_130_Peptostreptococcus	Firmicutes	0.270	0.120	0.135, 0.006–1.128▲	0.098, 0.000–0.664
OTU_133_Turicibacter	Firmicutes	0.070	0.040	0.043, 0.000–0.418▲	0.020, 0.000–0.141
OTU_348_Phyllobacteriaceae	Proteobacteria	0.040	0.080	0.021, 0.000–0.181▲	0.010, 0.000–0.317
OTU_593_Aeromicrobium	Actinobacteria	0.020	0.020	0.007, 0.000–0.128▲	0.000, 0.000–0.094
OTU_946_Clostridiaceae	Firmicutes	0.020	0.010	0.012, 0.000–0.063▲	0.000, 0.000–0.066
OTU_1020_Kineococcus	Actinobacteria	0.010	0.020	0.007, 0.000–0.035▲	0.000, 0.000–0.106
OTU_1078_Massilia	Proteobacteria	0.040	0.020	0.029, 0.000–0.134▲	0.009, 0.000–0.081
OTU_1159_Rubellimicrobium	Proteobacteria	0.080	0.060	0.052, 0.000–0.222▲	0.019, 0.000–0.239
OTU_1292_Pedobacter	Bacteroidetes	0.010	0.000	0.005, 0.000–0.054▲	0.000, 0.000–0.016
OTU_1469_Sarcina	Firmicutes	0.020	0.020	0.014, 0.000–0.188▲	0.005, 0.000–0.185
OTU_1621_Anaerococcus	Firmicutes	0.010	0.010	0.008, 0.000–0.087▲	0.000, 0.000–0.080
OTU_1674_Schlegelella	Proteobacteria	0.010	0.010	0.014, 0.000–0.054▲	0.000, 0.000–0.040
OTU_1763_Sanguibacter	Actinobacteria	0.020	0.030	0.019, 0.000–0.126▲	0.009, 0.000–0.275
OTU_32_Propionibacterium	Actinobacteria	7.690	10.350	1.419, 0.217–48.211	2.955, 0.188–51.543▲
OTU_1239_Thermomonas	Proteobacteria	0.040	0.030	0.015, 0.000–0.180	0.021, 0.000–0.199▲
OTU_1_Enterobacteriaceae	Proteobacteria	0.830	1.200	0.126, 0.000–4.800	0.278, 0.000–4.277▲
OTU_19_Xanthomonadaceae	Proteobacteria	0.290	0.480	0.106, 0.029–0.976	0.256, 0.000–1.837▲
OTU_1577_Sphingomonas	Proteobacteria	0.190	0.430	0.079, 0.006–1.068	0.122, 0.010–1.789▲
OTU_1670_Roseomonas	Proteobacteria	0.010	0.020	0.000, 0.000–0.144	0.009, 0.000–0.107▲
OTU_57_Chryseobacterium	Bacteroidetes	0.420	0.680	0.291, 0.068–1.941	0.437, 0.011–1.692▲
OTU_85_Roseomonas	Proteobacteria	0.020	0.030	0.008, 0.000–0.053	0.024, 0.000–0.127▲
OTU_111_Streptococcus	Firmicutes	0.120	0.250	0.091, 0.000–0.476	0.180, 0.000–1.035▲
OTU_115_Bacilli	Firmicutes	0.240	0.340	0.029, 0.000–1.582	0.083, 0.000–1.175▲
OTU_423_TM7_genera_incertae_sedis	TM7	0.030	0.020	0.011, 0.000–0.117	0.029, 0.000–0.077▲
OTU_545_Cellvibrio	Proteobacteria	0.010	0.020	0.000, 0.000–0.026	0.007, 0.000–0.183▲

A list of differentially abundant OTUs between lesional (Vitiligo) and non-lesional (Normal) skin obtained using the Wilcoxon test coupled to a bootstrapping approach. In each iteration of the bootstrap method, taxa with significantly different abundance were initially identified using Benjamini-Hochberg p-value correction at an FDR of 0.0001. Subsequently, taxa which were observed as having a significantly different abundance (post BH correction) in at least 99.5% of iterations were retained.
