# Supplementary material for: Microbial community profiling shows dysbiosis in the lesional skin of Vitiligo subjects
Source: Sci Rep. 2016 Jan 13;6:18761. doi: 10.1038/srep18761 (PMC4725359; doi:10.1038/srep18761)
Supplement: Supplementary Information [file srep18761-s1.pdf]

Microbial community profiling shows dysbiosis in the lesional skin of Vitiligo subjects

Parul Ganju[1,2#], Sunil Nagpal[3#], Mohammed MH[3], Nishal Kumar P[3], Rajesh Pandey[4], Vivek T Natarajan[1], Sharmila S. Mande[3*] and Rajesh S. Gokhale[1, 2, 5*]

Supplementary information content

Supplementary methods and references

Supplementary Figure S1: Alpha rarefaction plot for the samples used in the study
Supplementary Figure S2: Core skin microbiome of lesional and non-lesional samples
Supplementary Figure S3: Principal component analysis (PCoA) of lesional and non-lesional bacterial communities
Supplementary Figure S4: Intra-community network analysis: a) Non-lesional skin, b) Lesional Skin
Supplementary Figure S5: Sampling locations from Vitiligo subjects used in this study
Supplementary Figure S6: Schematic description of the algorithm used for taxonomic assignment of individual OTU clusters
Supplementary Figure S7: Community composition of lesional and non-lesional skin of Vitiligo subjects. Samples were obtained from anatomically similar sites.
Supplementary Figure S8: α-diversity trends in lesional (Vitiligo) and non-lesional (Normal) skin samples taken from anatomically similar sites
Supplementary Figure S9: Core microbiota comparison between lesional and non-lesional skin of vitiligo subjects. The samples were taken from anatomically similar sites.
Supplementary Figure S10: Ordination Analysis of cutaneous microbiome of lesional and non-lesional skin. The samples were taken from anatomically similar sites
Supplementary Figure S11: Differentiating taxa of lesional and non-lesional skin. The samples were taken from anatomically similar sites.

Microbial community profiling shows dysbiosis in the lesional skin of Vitiligo subjects

Parul Ganju[1,2#], Sunil Nagpal[3#], Mohammed MH[3], Nishal Kumar P[3], Rajesh Pandey[4], Vivek T Natarajan[1], Sharmila S. Mande[3*] and Rajesh S. Gokhale[1, 2, 5*]

Supplementary information content *(cont.)*

Supplementary Figure S12: Comparison between a) non-lesional and (b) lesional bacterial composition using Intra-community network analysis. The samples were taken from anatomically similar sites.
Supplementary Figure S13: Intra-community network analysis of cutaneous microbiota. The samples were taken from anatomically similar sites.

Supplementary Table S1: Good's coverage estimate for sampling completeness for all samples
Supplementary Table S2: Summary of 'number of taxa' at various ranges of relative contribution
Supplementary Table S3: Comparison of properties of lesional and non-lesional microbial community networks
Supplementary Table S4: Comparison of various Phylogenetic and Phenotypic properties of top degree nodes of the microbial community interaction network for a) Non-lesional and (b) Lesional datasets of present study.
Supplementary Table S5: Subject information
Supplementary Table S6: MID sequences used for primer designing
Supplementary Table S7: Sample Ids, sampling sites and primers used for each sample in this study
Supplementary Table S8: A list of differentially abundant taxa (RDP classification) between lesional and non-lesional skin obtained using the Wilcoxon test coupled to a bootstrapping approach. Samples were obtained from anatomically similar sites.
Supplementary Table S9: A list of differentially abundant OTUs between lesional (Vitiligo) and non-lesional (Normal) skin obtained using the Wilcoxon test coupled to a bootstrapping approach. Samples were obtained from anatomically similar sites.

Supplementary methods

Sample Collection

Samples were collected from non-overlapping sites, with no prior cleaning of skin surface by swabbing[1]. Swabs were obtained from 5 cm^2 areas using sterile cotton tipped applicators (HiMedia) soaked in enzymatic lysis buffer (sterile 0.15 M NaCl with 0.1% Tween 20). Negative controls of mock swabs were collected and analyzed for each sampling. Paired samples were collected from lesional (depigmented) and non-lesional (uninvolved pigmented) skin sites from each subject. Samples of the uninvolved skin were taken close to the lesional skin (about 10 cm distance).

Sequencing coverage, Rarefaction analysis and Diversity indices

Sequencing coverage was estimated using Good's index[2]. Rarefaction analysis was also performed on the raw-OTU cluster abundance data using PAST 3.0[3]. The R package phyloseq [4] was employed on (1) genus level abundance data obtained using RDP classifier, and (2) OTU level abundance data for calculating various microbial diversity indices.

References

1. Grice, E.A. et al. Topographical and temporal diversity of the human skin microbiome. Science 324, 1190-2 (2009).
2. Good, I.J. The population frequencies of species and the estimation of population parameters. Biometrika 40, 237–264 (1953).
3. Hammer, O., Harper, D.A.T., Ryan, P.D. PAST: Paleontological statistics software package for education and data analysis. Palaeontologia Electronica 4, 9pp (2001).
4. McMurdie, P.J. & Holmes, S. phyloseq: an R package for reproducible interactive analysis and graphics of microbiome census data. *PLoS One* **8**, e61217 (2013).

Alpha rarefaction plot for the samples used in the study

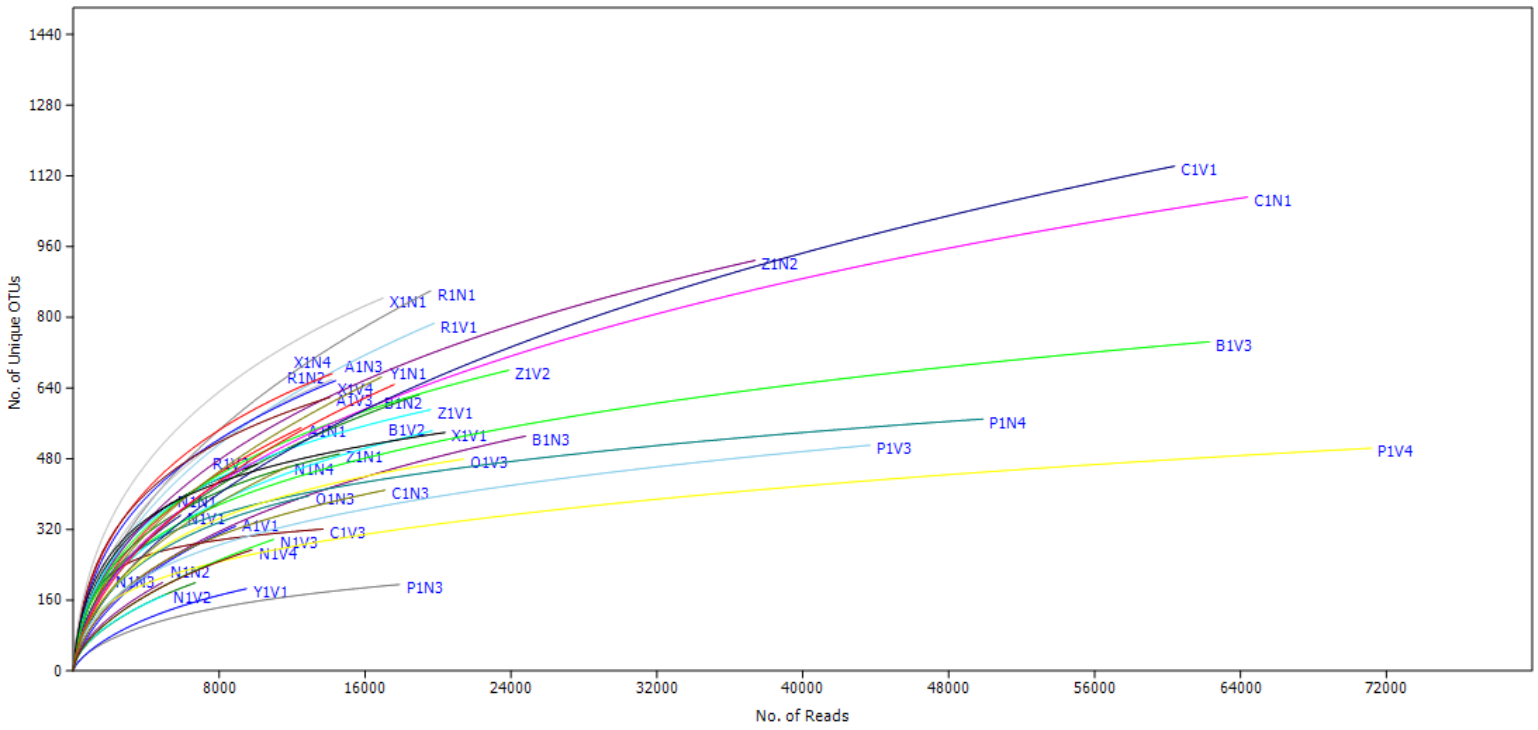

Figure S1: Rarefaction plot for all 40 samples of the present study, indicating reasonable coverage for most samples.

Supplementary Figure S1

Core skin microbiome of lesional and non-lesional samples

a).

Core Genera (RDP)	Bootstrap Score*	
	Normal	Vitiligo
Methylobacterium	12	72.2
Corynebacterium	100	100
Propionibacterium	100	100
Kocuria	100	100
Staphylococcus	100	100
Streptococcus	100	100
Streptophyta	72.2	36.6
Micrococcus	100	100
Anaerococcus	100	3.1
Acinetobacter	100	100
Prevotella	100	100
Pseudomonas	100	100
Chryseobacterium	71.7	100
Enhydrobacter	69.1	100
Dietzia	100	100
Microbacterium	70	11
Brachybacterium	100	100
Brevibacterium	100	100
Ornithinimicrobium	100	100
Janibacter	100	100
Nocardioides	69.3	36.2
Paracoccus	100	100
Brevundimonas	100	71.3

b).

Core OTUs	Bootstrap Score*	
	Normal	Vitiligo
OTU_1129_Pseudomonas	100	100
OTU_12_Paracoccus	100	100
OTU_1652_Dietzia	100	100
OTU_2_Micrococcus	100	100
OTU_26_Corynebacterium	100	100
OTU_27_Staphylococcus	100	100
OTU_29_Streptococcus	100	100
OTU_32_Propionibacterium	100	100
OTU_460_Kocuria	100	100
OTU_49_Ornithinimicrobium	100	100
OTU_57_Chryseobacterium	100	100
OTU_61_Brachybacterium	100	100
OTU_9_Acinetobacter	100	100
OTU_1641_Kocuria	100	69.1
OTU_195_Janibacter	69.2	0
OTU_50_Brevundimonas	68.2	0
OTU_116_Enhydrobacter	0	100
OTU_302_Paracoccus	0	100
OTU_95_Staphylococcus	0	100

**Bootstrap Score (0-100); s=15; n=1000*
s: No. of samples randomly picked
n: Number of iterations performed

Figure S2: Comparison of core-microbial (a) genera and (b) OTUs between Non-Lesional and Lesional samples. Identification of core taxa (genera/OTUs) was done using a bootstrapping approach, wherein a random subset (i.e. 75% of samples) were drawn from the whole set of samples, and core microbial taxa were deduced from the randomly drawn sample set. This approach was iterated 1000 times to arrive at 1000 sets of core microbial taxa for the whole sample set. A union of core taxa for all 1000 iterations was generated and a bootstrap score was assigned to each taxon based upon it's consistency in appearing as core taxon in all iterations. Bootstrap score was scaled between 0-100, wherein a score of 100 for a taxon indicated that the given taxon appeared as core in all 1000 iterations. In this study, only those taxa were considered as core which had minimum bootstrap score of 50. In this figure, each bar (blue, red) signifies the bootstrap score for the corresponding genus/OTU identified as core. (Blue: Normal/Non-lesional and Red: Vitiligo/Lesional)

Supplementary Figure S2

Principal component analysis (PCoA) of lesional and non-lesional bacterial communities

a). b).

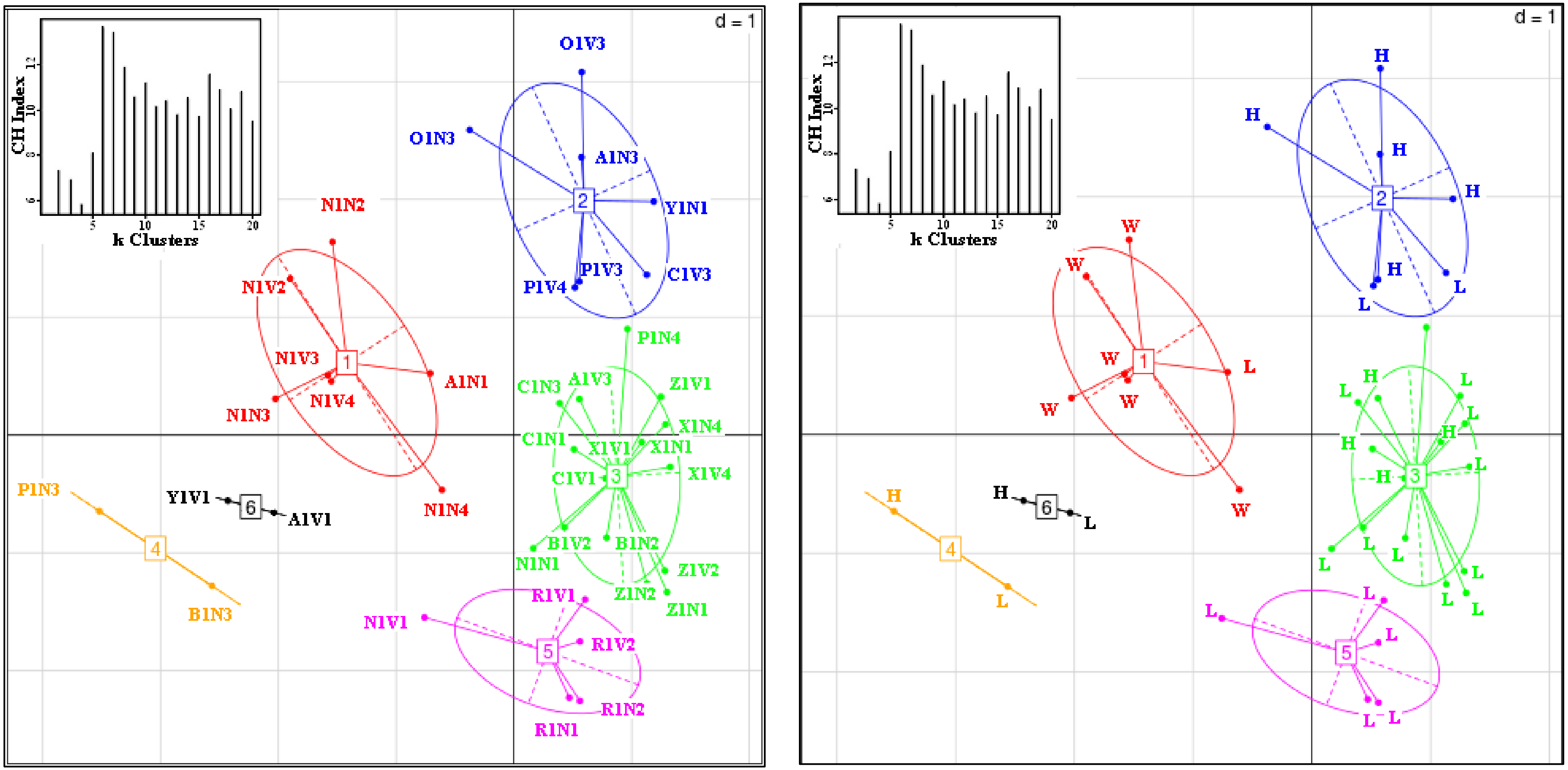

Figure S3: a) PCoA analysis visualizing the results from PCA and clustering of Non-Lesional (N) and Lesional (V) samples. (b) PCoA analysis visualizing the results from PCA and clustering of all samples of the present study, tagged according to the site of sampling. Samples were clustered using Jensen–Shannon distance and partitioning around medoid (PAM) clustering. Inset represents the results of optimal number of clusters estimation based on Calinski–Harabasz (CH) index.

Supplementary Figure S3

Intra-community network analysis: Non-lesional skin

a).

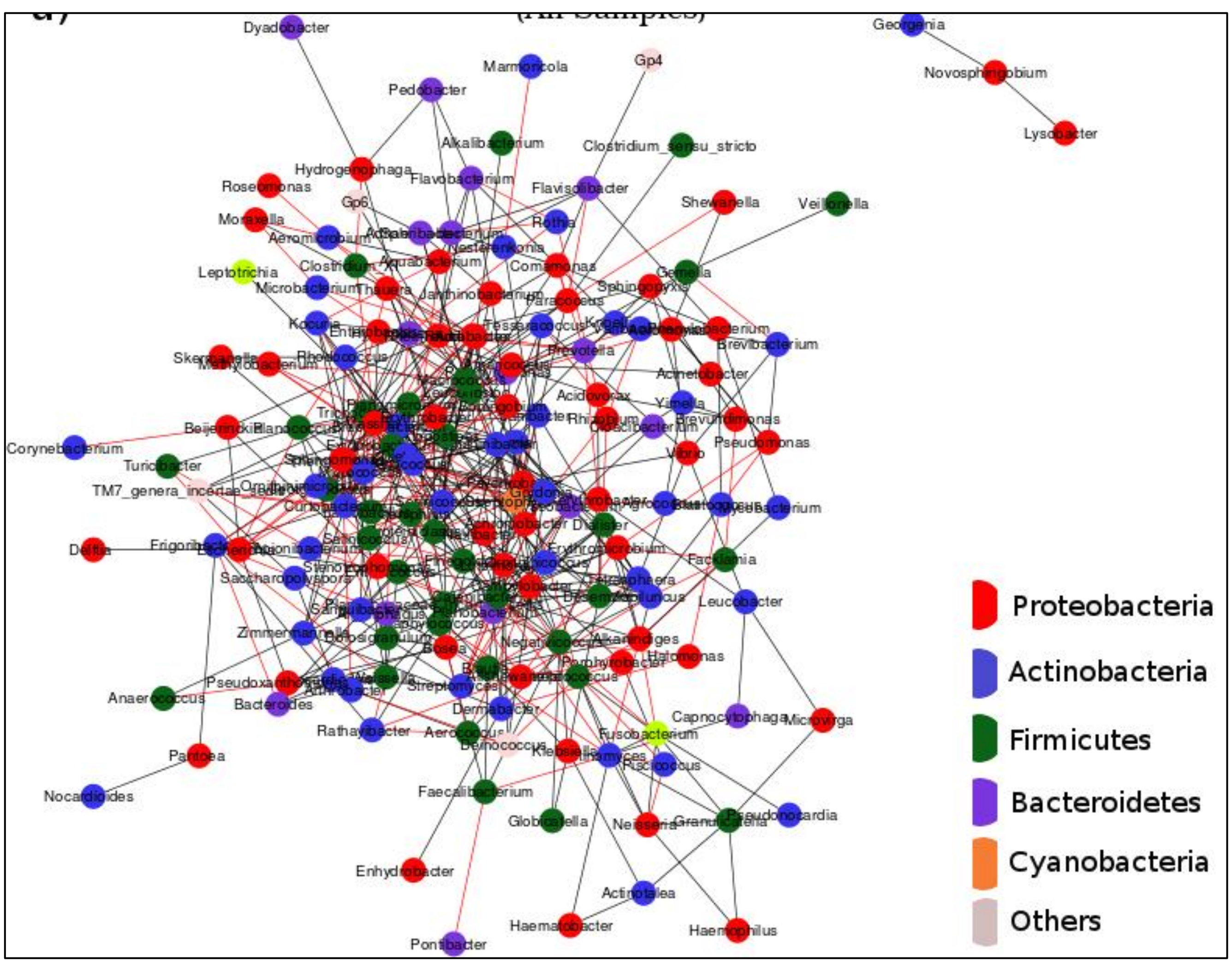

Figure S4a: Spearman correlation based micorbial communtiy interaction network generated for non-lesional samples. Nodes have been colored according to the phylum level affiliation of the network members, wherein each network members represents a microbial genus.

Supplementary Figure S4

b).

Intra-community network analysis: Lesional skin

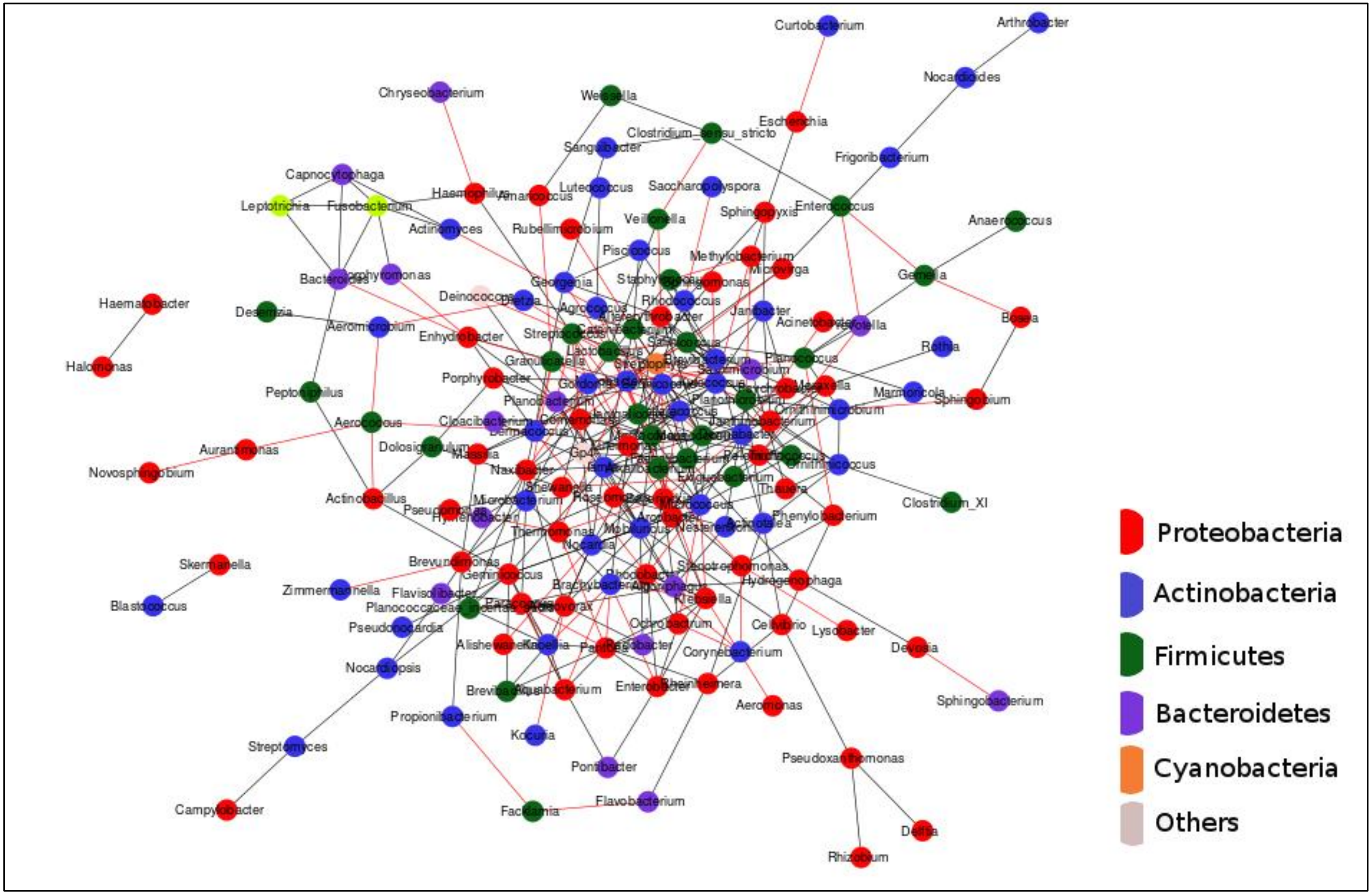

Figure S4b: Spearman correlation based micorbial communtiy interaction network generated for lesional samples. Nodes have been colored according to the phylum level affiliation of the network members, wherein each network members represents a microbial genus.

Supplementary Figure S4

Sampling locations from Vitiligo subjects used in this study

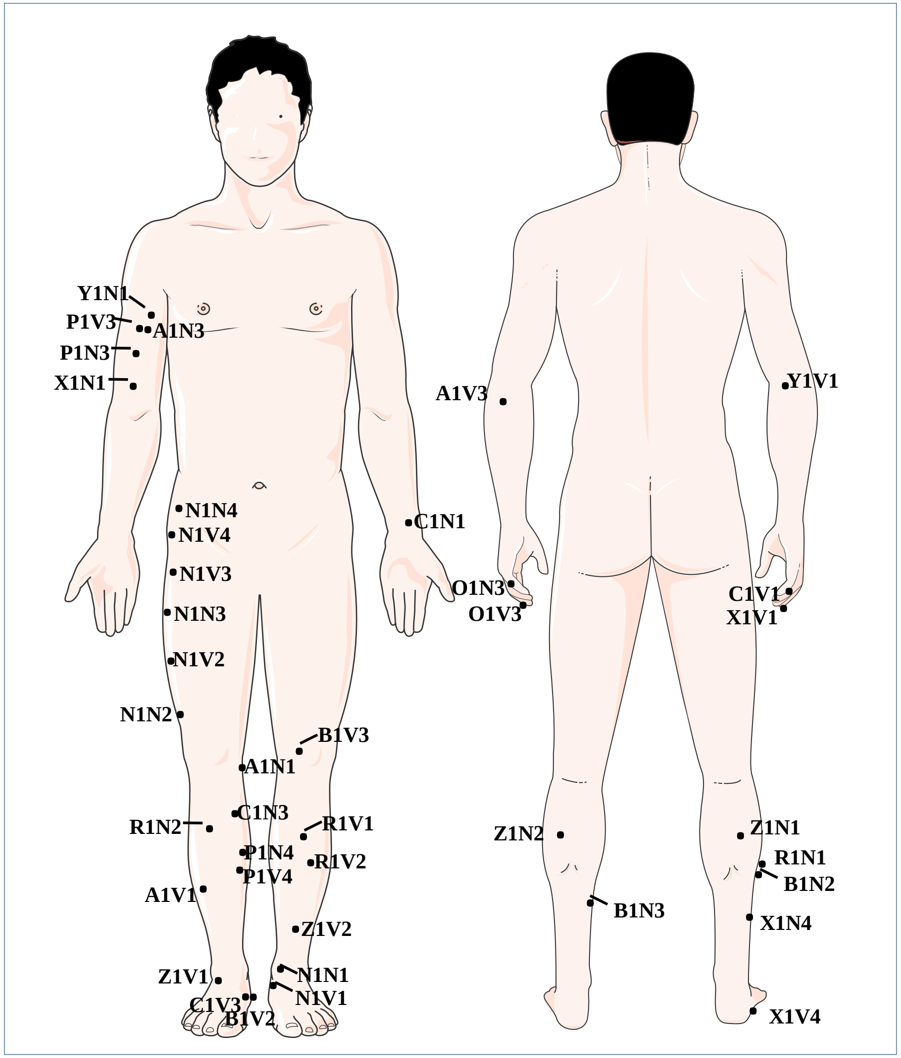

Figure S5: Various sampling locations along with corresponding identifier tags that have been referred to in this study/document. Sample nomenclature was as follow: **Subject ID-involved versus uninvolved site-site number**. For example, A1N1 means sample has been obtained from non-lesional (normal) skin of subject A1 at site 1. Corresponding sample from lesional (vitiliginous) skin close to site 1 would be A1V1. (Image adapted from Sevier Medical Art Tools: http://www.servier.com/Powerpoint-image-bank)

Supplementary Figure S5

Schematic description of the algorithm used for Taxonomic assignment of individual OTU clusters

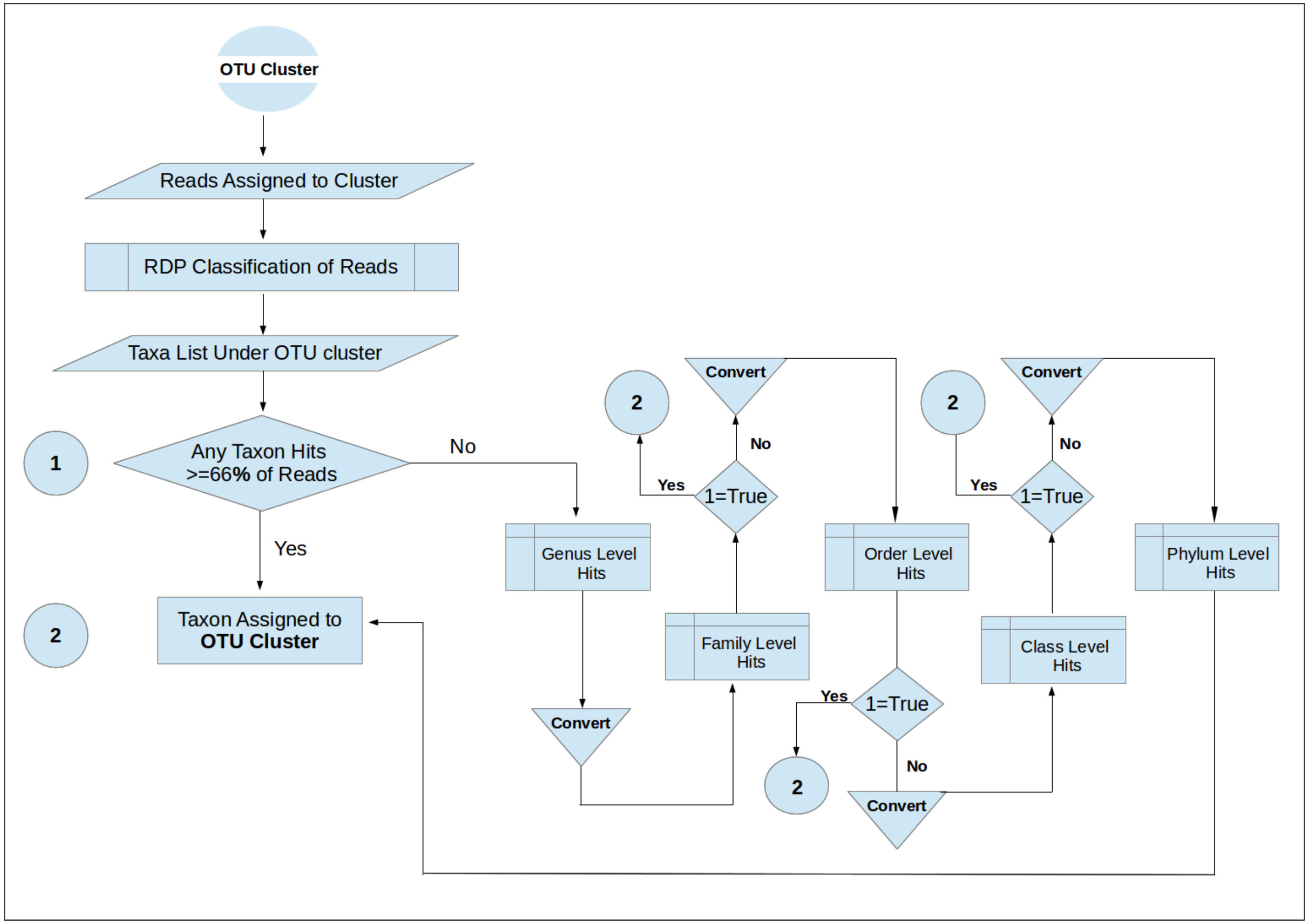

Figure S6: The assignment process proceeds in the following manner. If greater than two-thirds of reads in a cluster had an identical taxonomic assignment (i.e. classified by RDP to the same taxon), the OTU cluster is assigned to that taxon. In the event of any taxon in a cluster not attaining a cumulative percentage of 66%, all genus level assignments in that cluster are re-assigned to their immediately higher taxonomic level (i.e. corresponding family level), and the process of checking if any taxon exceeded 66% is repeated. This process is iterated until all clusters obtain a taxonomic assignment

Supplementary Figure S6

Community composition of lesional and non-lesional skin of Vitiligo subjects. Samples were obtained from anatomically similar sites.

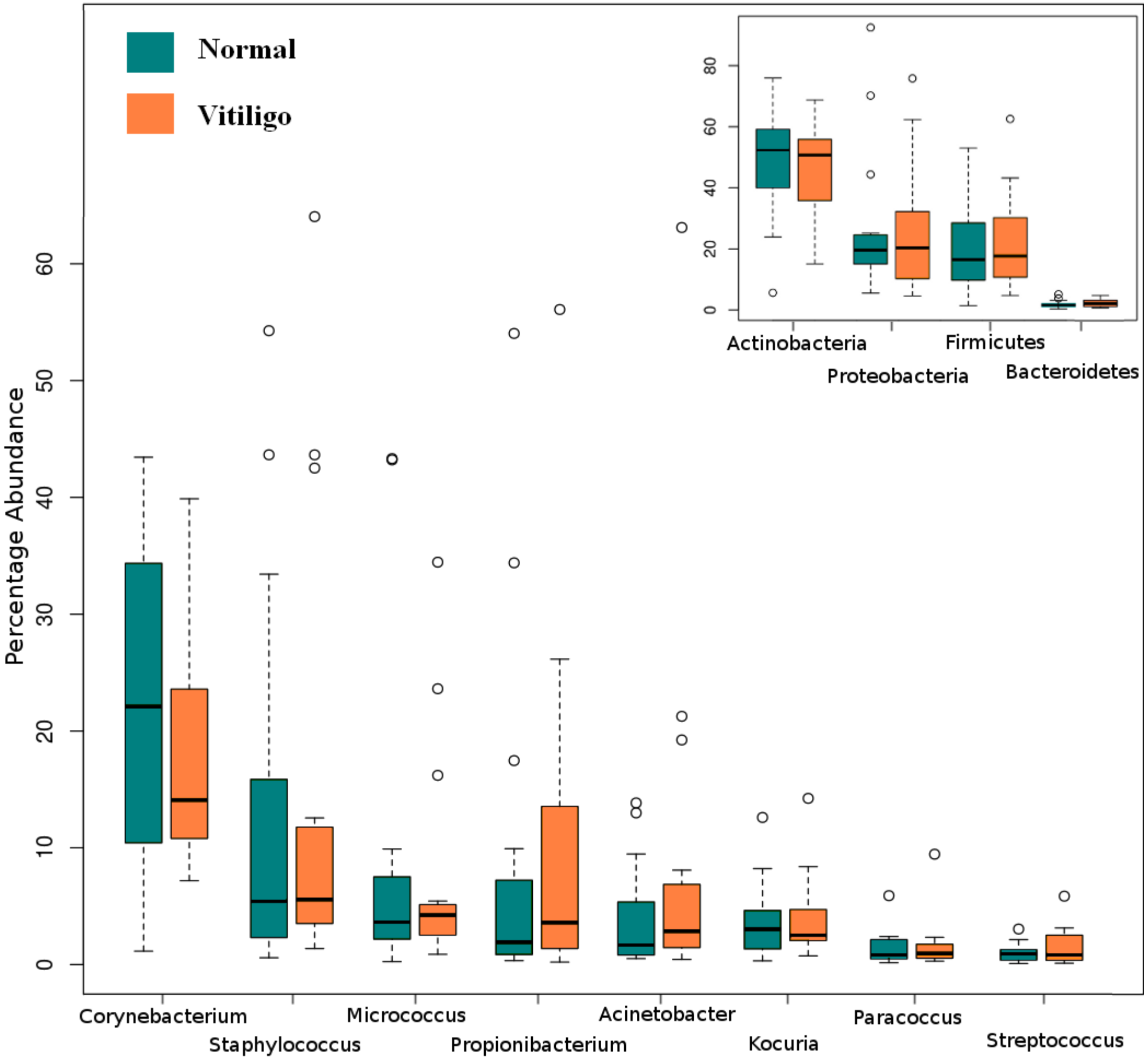

Figure S7: Box-plots representing relative abundance analysis of the bacterial taxa discovered in samples obtained from anatomically similar Non-Lesional (Normal) and Lesional (Vitiligo) sites at genus (main) and phylum (inset) levels. Taxa with minimum median abundance of 1% were used for the comparison. The results depicted in the above figure indicate that the microbial abundances observed in the subset of samples (30) from anatomically similar skin sites are similar to that observed during analysis of the whole sample set (40 samples).

Supplementary Figure S7

α-diversity trends in lesional (Vitiligo) and non-lesional (Normal) skin samples taken from anatomically similar sites

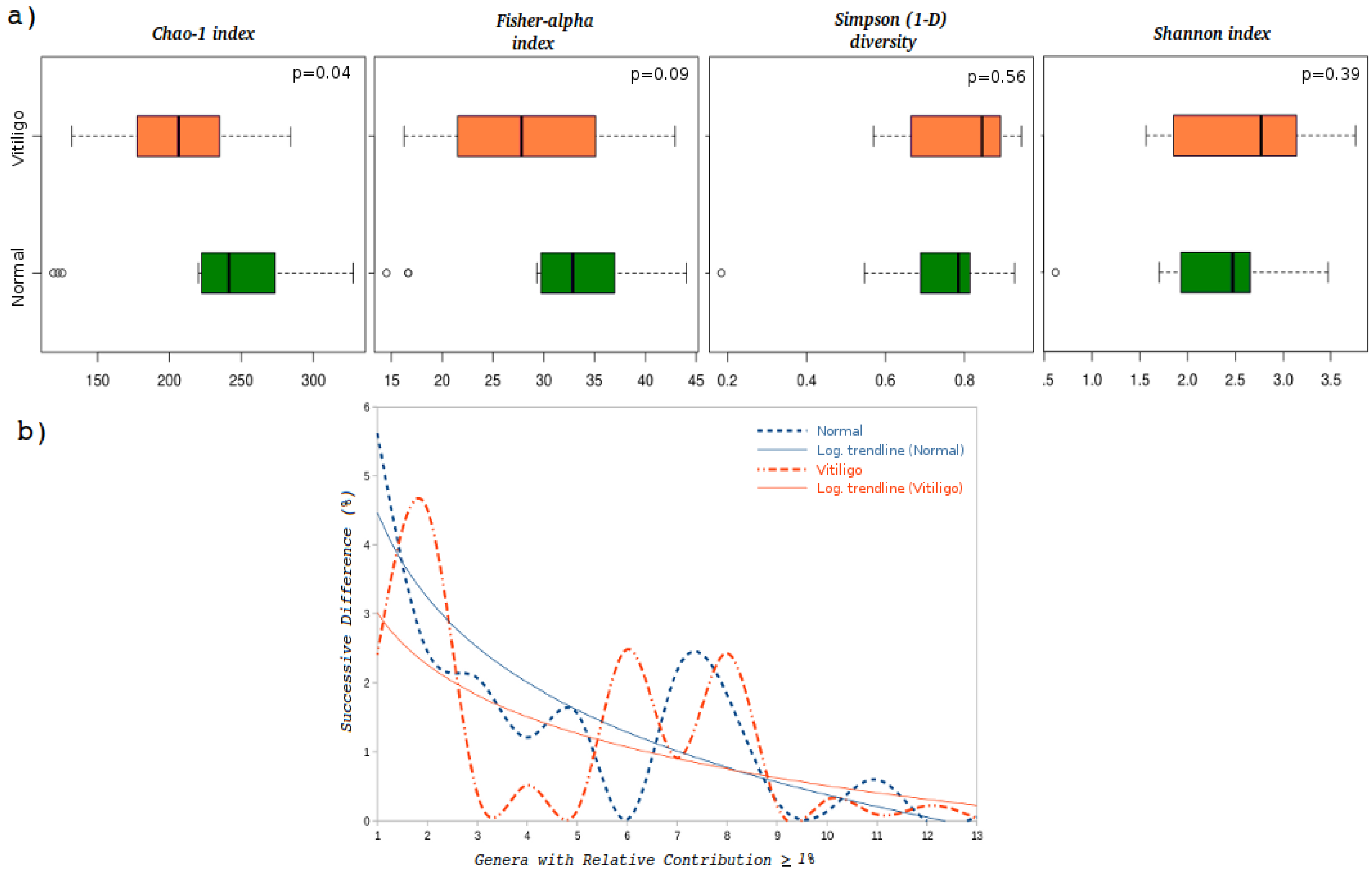

Figure S8: a) Box-plots illustrating the comparison of diversity indices (Chao-1, Fisher, Simpson1-D and Shannon index) between Non-Lesional and Lesional samples from anatomically similar sites.

b) Comparison of differences between successive relative contribution values (of the ordered genera) in Non-Lesional and Lesional samples. Only those genera were considered for calculating successive differences in relative contributions that had a minimum relative contribution of 1%. Consistent with the previously observed results for overall population, diversity analysis using the subset of samples from anatomically similar sites indicate similar pattern with respect to various diversity metrics.

Supplementary Figure S8

Core microbiota comparison between lesional and non-lesional skin of vitiligo subjects. The samples were taken from anatomically similar sites.

a)

Core OTUs	Bootstrap score *	
	Normal	Vitiligo
OTU_2_Micrococcus	100	100
OTU_9_Acinetobacter	100	100
OTU_12_Paracoccus	100	100
OTU_26_Corynebacterium	100	100
OTU_27_Staphylococcus	100	100
OTU_29_Streptococcus	100	100
OTU_32_Propionibacterium	100	100
OTU_49_Ornithinimicrobium	100	100
OTU_57_Chryseobacterium	100	100
OTU_61_Brachybacterium	100	100
OTU_95_Staphylococcus	100	100
OTU_460_Kocuria	100	100
OTU_1129_Pseudomonas	100	100
OTU_1641_Kocuria	100	100
OTU_1652_Dietzia	100	100
OTU_302_Paracoccus	62.9	100
OTU_662_Brachybacterium	62	62.8
OTU_50_Brevundimonas	60.7	64.6
OTU_195_Janibacter	62.9	0
OTU_116_Enhydrobacter	0	100
OTU_38_Intrasporangiaceae	0	64
OTU_160_Porphyrobacter	0	61

b)

Core Genera (RDP)	Bootstrap score *	
	Normal	Vitiligo
Kocuria	100	100
Micrococcus	100	100
Ornithinimicrobium	100	100
Paracoccus	100	100
Prevotella	100	100
Propionibacterium	100	100
Pseudomonas	100	100
Staphylococcus	100	100
Streptococcus	100	100
Chryseobacterium	100	100
Corynebacterium	100	100
Dietzia	100	100
Brachybacterium	100	100
Brevibacterium	100	100
Acinetobacter	100	100
Janibacter	100	65.7
Brevundimonas	64.3	63.9
Enhydrobacter	0	100
Anaerococcus	64.1	0

Bootstrap Score (0-100); s=11; n=1000
s: No. of samples randomly picked
n: Number of iterations performed

Figure S9: Comparison of core-microbial (a) OTUs and (b) RDP genera between Non-Lesional (Normal) and Lesional (Vitiligo) samples taken from anatomically similar sites. Identification of core taxa (genera/OTUs) was done using the bootstrapping approach. Normal and Vitiligo samples pertaining to anatomically similar sites were observed to share a fairly common core taxa profile (18 OTUs and 17 RDP genera constituting the common core). OTUs pertaining to Enhydrobacter (OTU-116), Intrasporangiaceae (OTU-38), and Porphyrobacter (OTU-160) were observed to be the 'Core' set exclusive to Vitiligo samples. The RDP taxon corresponding to Enhydrobacter was observed to be a genus that is 'Core' exclusively in Vitiligo samples. The results with the samples corresponding to anatomically similar sites were observed to have good similarity with results obtained with the complete set of samples.

Supplementary Figure S9

Ordination Analysis of cutaneous microbiome of lesional and non-lesional skin. The samples were taken from anatomically similar sites

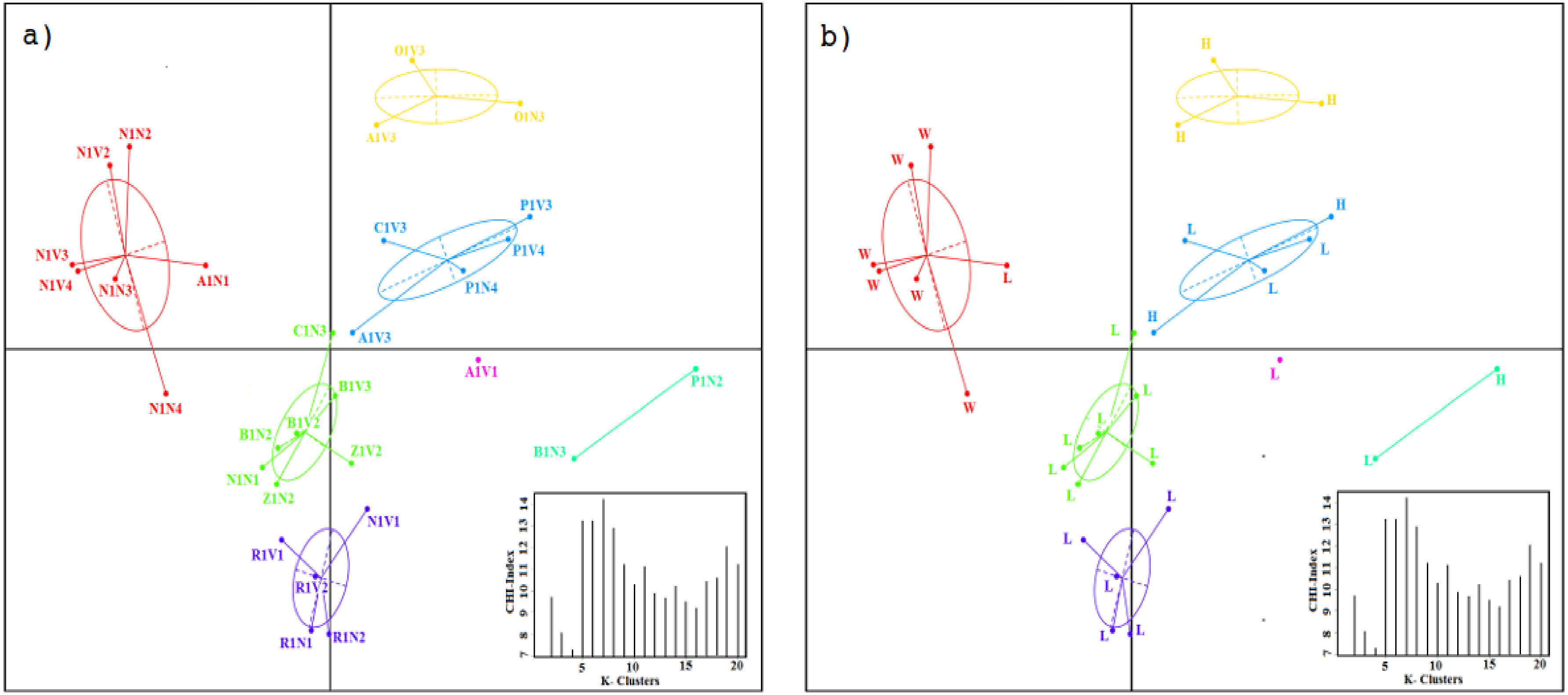

Figure S10: a) PCoA analysis visualizing the results from PCA and clustering of Non-Lesional (N) and Lesional (V) samples taken from anatomically similar sites. (b) PCoA analysis visualizing the results from PCA and clustering of samples taken from anatomically similar sites tagged according to the site of sampling. Samples were clustered using Jensen–Shannon distance and partitioning around medoid (PAM) clustering. Inset represents the results of optimal number of clusters estimation based on Calinski–Harabasz (CH) index.

As seen in the earlier results obtained with the complete set of samples, oridination analysis performed with the subset of samples from anatomically similar sites also indicates a clustering pattern based on subjects rather than disease status thereby suggesting individual-specific microbiome signatures

Supplementary Figure S10

Differentiating taxa of lesional and non-lesional skin. The samples were taken from anatomically similar sites.

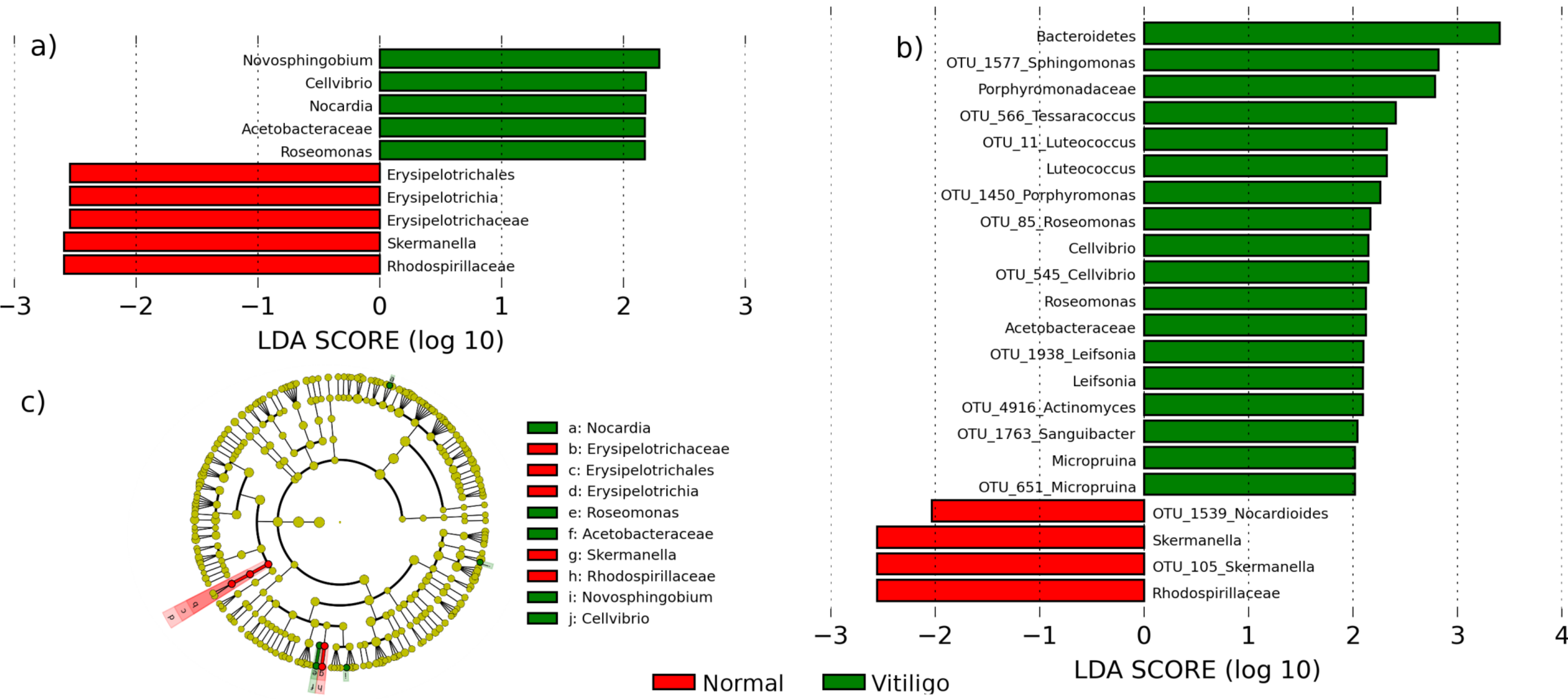

Figure S11: Comparison of LDA effect size of the significantly differentiating microbial taxa (a) RDP level and (b) OTU level between Non-Lesional and Lesional samples. LefSe package was used to generate the LDA effect size with LDA cut-off = 2. Wilcoxon p value cut-off of 0.1 was used for differentiating feature analysis through LefSe.. c) Cladogram illustrating the phylogenetic relationship amongst the significantly differentiating microbial taxa (RDP level) between Non-Lesional and Lesional samples, deduced using LefSe package.

Supplementary Figure S11

Comparison between lesional and non-lesional bacterial composition using Intra-community network analysis. The samples were taken from anatomically similar sites.

a).

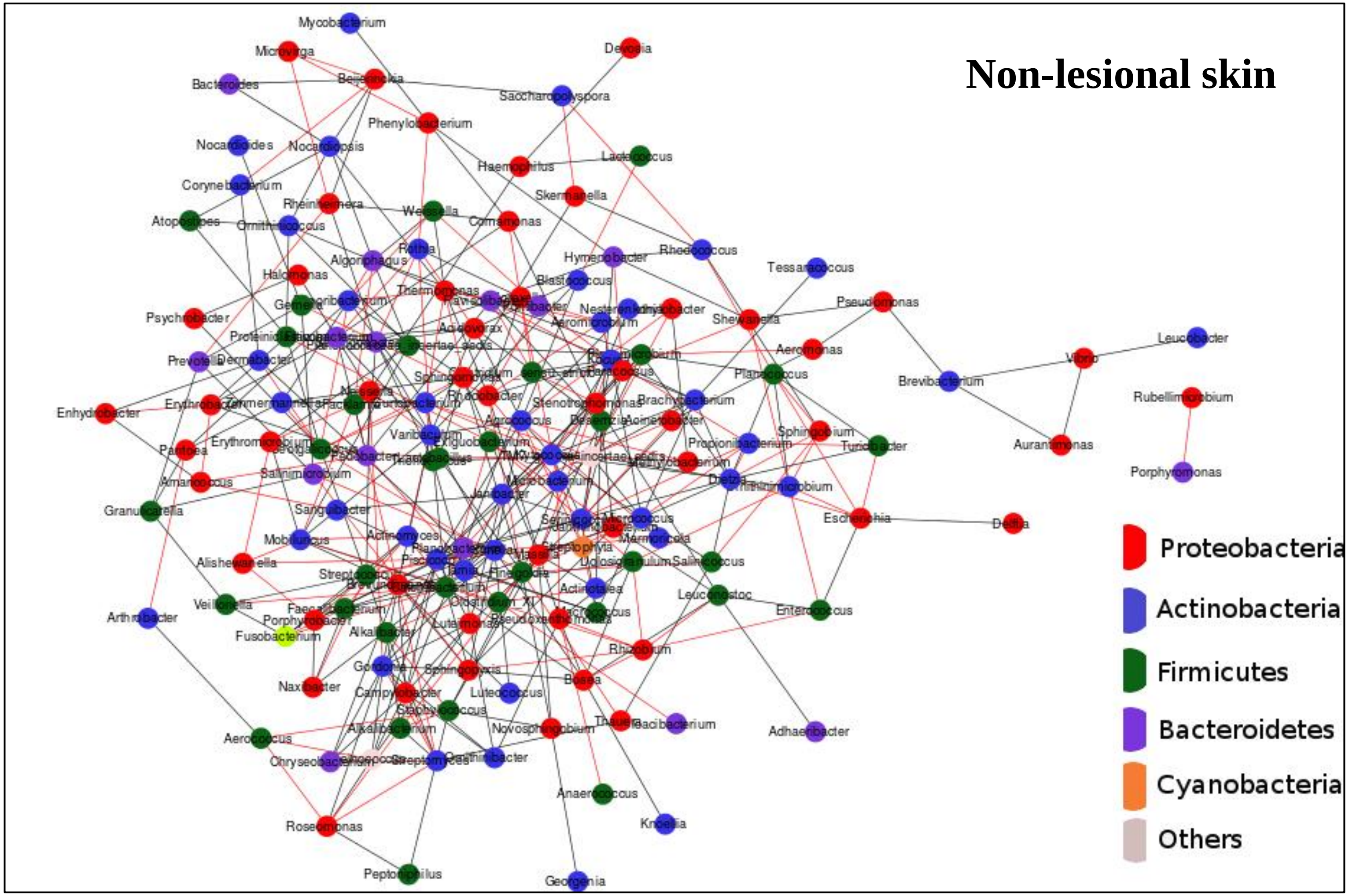

Figure S12a: Spearman correlation based micorbial communtiy interaction network generated for non-lesional samples. Nodes have been colored according to the phylum level affiliation of the network members, wherein each network members represents a microbial genus. Also refer to supplementary table S3b.

Supplementary Figure S12

Comparison between lesional and non-lesional bacterial composition using Intra-community network analysis. The samples were taken from anatomically similar sites.

b).

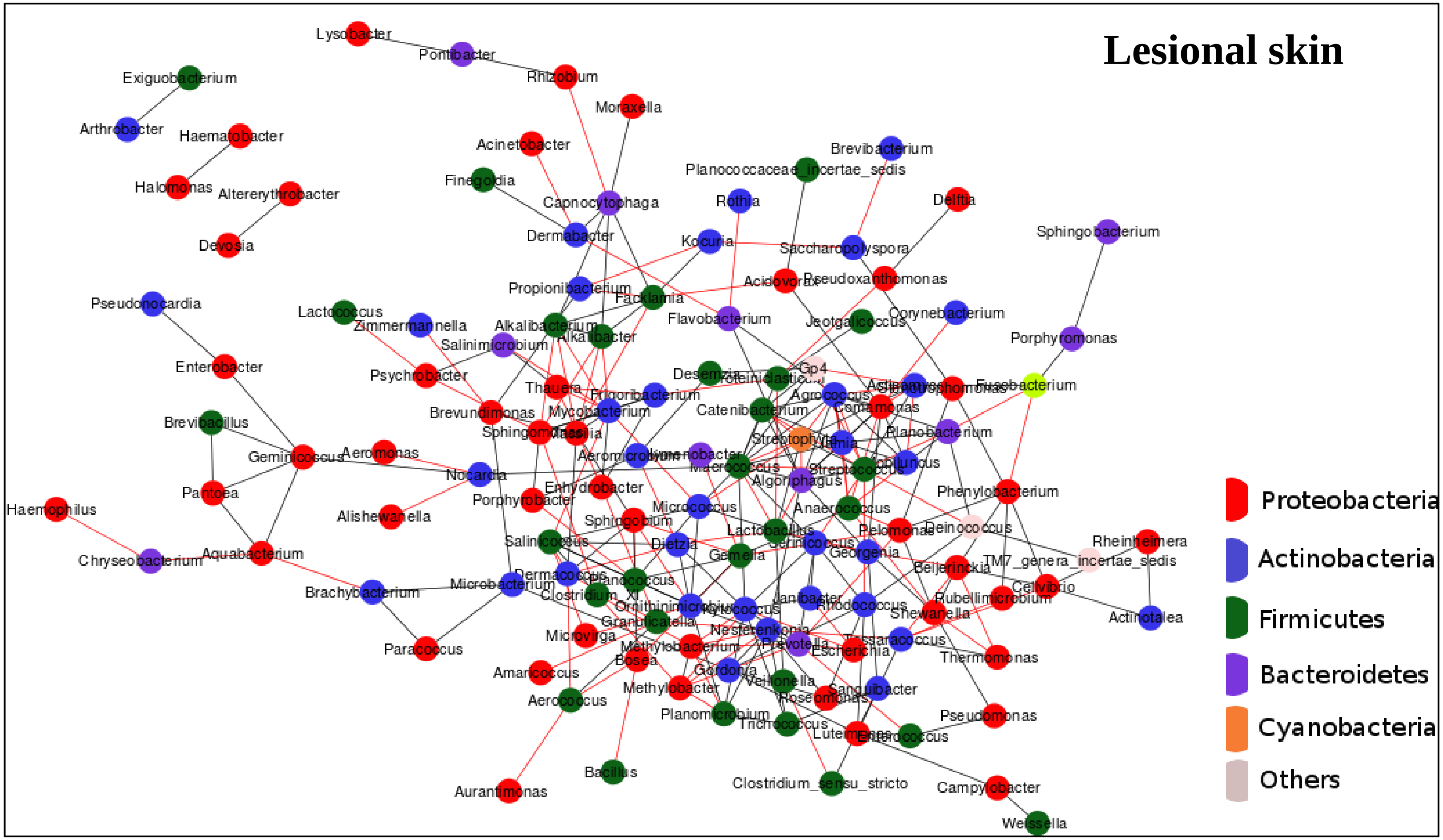

Figure S12b: Spearman correlation based micorbial communtiy interaction network generated for non-lesional samples. Nodes have been colored according to the phylum level affiliation of the network members, wherein each network members represents a microbial genus.

Supplementary Figure S12

Intra-community network analysis of cutaneous microbiota. The samples were taken from anatomically similar sites.

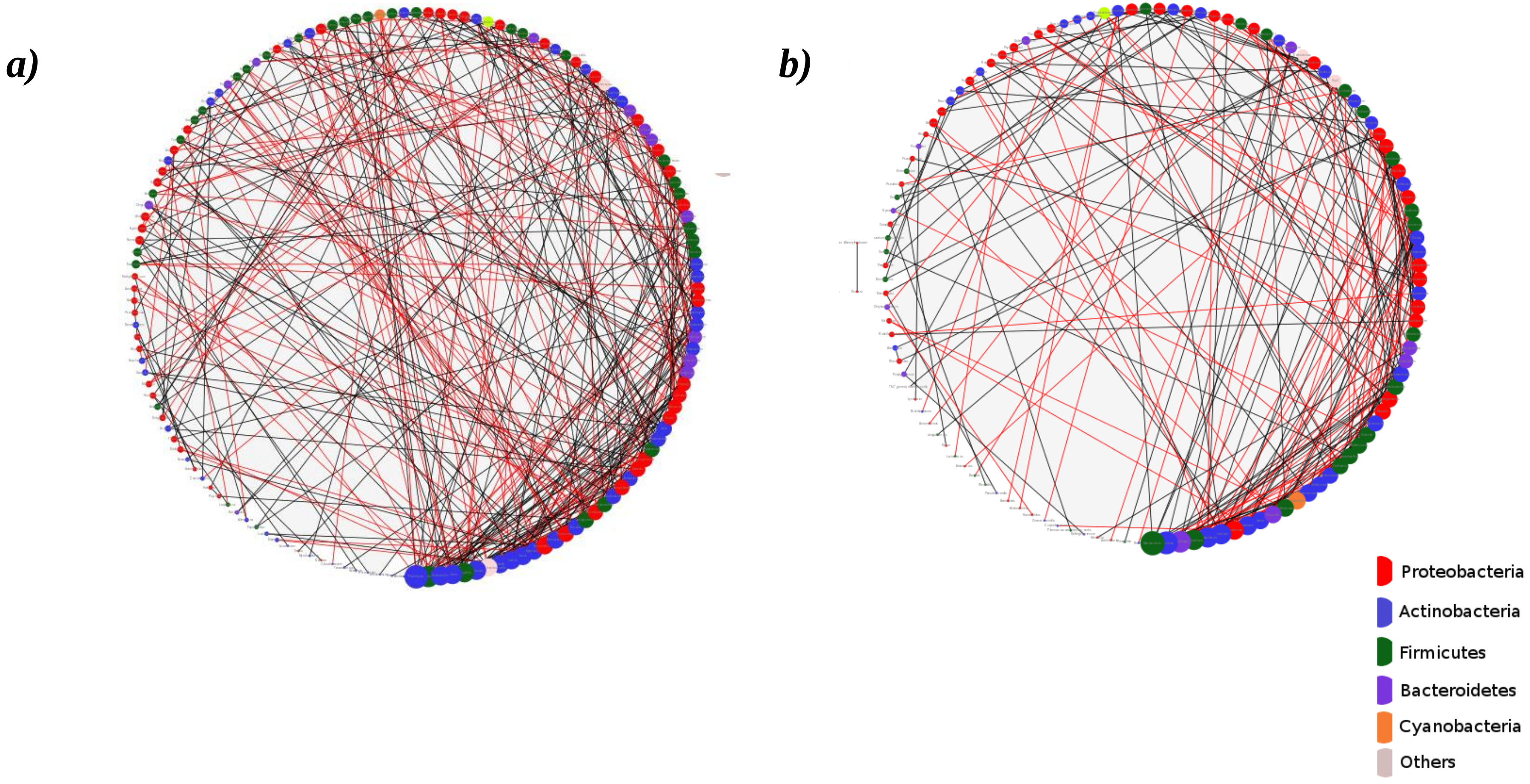

Figure S13 Degree sorted circular layout of the networks generated for (a) Non-Lesional and (b) Lesional sample sets taken from anatomically similar sites Nodes are sorted according to their degree in such a way that the size of the node serves as an index of the magnitude of its degree. Color of the nodes represents their phylum affiliation.

Supplementary Figure S13

Table S1: Good's coverage estimate for sampling completeness for all samples

Sample Name	Good's Coverage Value
A1N1	0.98
A1V1	0.982
B1N2	0.986
B1V2	0.99
B1N3	0.991
B1V3	0.996
C1N3	0.992
C1V3	0.997
N1N1	0.971
N1V1	0.973
P1N4	0.997
P1V4	0.998
R1N1	0.979
R1V1	0.982
R1N2	0.983
R1V2	0.986
X1N4	0.984
X1V4	0.983
Z1N1	0.99
Z1V1	0.991
Z1N2	0.991
Z1V2	0.99
A1N3	0.98
A1V3	0.987
C1N1	0.994
C1V1	0.992
O1N3	0.991
O1V3	0.994
P1N3	0.996
P1V3	0.996
X1N1	0.983
X1V1	0.994
Y1N1	0.982

Y1V1	0.991
N1N2	0.986
N1V2	0.983
N1N3	0.977
N1V3	0.986
N1N4	0.981
N1V4	0.987

Table S2: Summary of 'number of taxa' at various ranges of relative contribution

Relative Contribution (%)	No. of Genera (% w.r.t total no. of taxa)	
	Non-Lesional	Lesional
≥1.00	12 (1.9)	16 (2.8)
≥0.10	58 (9.5)	83 (14.3)
≥0.01	216 (35.3)	226 (39.0)

Relative Contribution (%)	No. of Families (% w.r.t total no. of taxa)	
	Non-Lesional	Lesional
≥1.00	12 (6.9)	18 (10.7)
≥0.10	55 (31.4)	59 (35.1)
≥0.01	113 (64.6)	114 (67.9)

Table S3: Comparison of properties of lesional and non-lesional microbial community networks. a) All samples, (b) Samples from anatomically similar sites

a)

Network Properties	Samples	
	**Non-Lesional**	**Lesional**
Nodes	162	151
Edges	511	348
Average Degree	6.27	4.58
Shortest Path Length	3.35	3.96
Clustering Coefficient	0.28	0.26

b)

Network Properties	Samples	
	**Non-Lesional**	**Lesional**
Nodes	148	127
Edges	407	259
Average Degree	5.46	4.05
Shortest Path Length	3.67	4.32
Clustering Coefficient	0.30	0.31

Table S4a: Comparison of various Phylogenetic and Phenotypic properties of top degree nodes of the microbial community interaction network for Non-lesional datasets of present study.

Phenotypes	**Degree Threshold**					
Phylum	**70.0**	**75.0**	**80.0**	**85.0**	**90.0**	**95.0**
Actinobacteria	46.7	45.5	42.9	50.0	66.7	100.0
Bacteroidetes	6.7	0.0	0.0	0.0	0.0	0.0
Firmicutes	26.7	27.3	42.9	50.0	33.3	0.0
Proteobacteria	20.0	27.3	14.3	0.0	0.0	0.0
Class	**70.0**	**75.0**	**80.0**	**85.0**	**90.0**	**95.0**
Actinobacteria	46.7	45.5	42.9	50.0	66.7	100.0
Alphaproteobacteria	6.7	9.1	14.3	0.0	0.0	0.0
Bacilli	13.3	18.2	28.6	25.0	0.0	0.0
Epsilonproteobacteria	6.7	9.1	0.0	0.0	0.0	0.0
Erysipelotrichia	6.7	9.1	14.3	25.0	33.3	0.0
Flavobacteria	6.7	0.0	0.0	0.0	0.0	0.0
Gammaproteobacteria	6.7	9.1	0.0	0.0	0.0	0.0
Negativicutes	6.7	0.0	0.0	0.0	0.0	0.0
Order	**70.0**	**75.0**	**80.0**	**85.0**	**90.0**	**95.0**
Acidimicrobiales	6.7	9.1	14.3	25.0	33.3	50.0
Actinomycetales	40.0	36.4	28.6	25.0	33.3	50.0
Bacillales	6.7	9.1	14.3	0.0	0.0	0.0
Campylobacterales	6.7	9.1	0.0	0.0	0.0	0.0
Erysipelotrichales	6.7	9.1	14.3	25.0	33.3	0.0
Flavobacteriales	6.7	0.0	0.0	0.0	0.0	0.0
Lactobacillales	6.7	9.1	14.3	25.0	0.0	0.0
Selenomonadales	6.7	0.0	0.0	0.0	0.0	0.0
Sphingomonadales	6.7	9.1	14.3	0.0	0.0	0.0
Xanthomonadales	6.7	9.1	0.0	0.0	0.0	0.0
Family	**70.0**	**75.0**	**80.0**	**85.0**	**90.0**	**95.0**
Campylobacteraceae	6.7	9.1	0.0	0.0	0.0	0.0
Carnobacteriaceae	6.7	9.1	14.3	25.0	0.0	0.0
Dermabacteraceae	6.7	9.1	14.3	25.0	33.3	50.0
Dermacoccaceae	6.7	9.1	14.3	0.0	0.0	0.0
Dietziaceae	6.7	9.1	0.0	0.0	0.0	0.0
Erysipelotrichaceae	6.7	9.1	14.3	25.0	33.3	0.0
Flavobacteriaceae	6.7	0.0	0.0	0.0	0.0	0.0
Iamiaceae	6.7	9.1	14.3	25.0	33.3	50.0
Intrasporangiaceae	13.3	9.1	0.0	0.0	0.0	0.0
Microbacteriaceae	6.7	0.0	0.0	0.0	0.0	0.0
Sphingomonadaceae	6.7	9.1	14.3	0.0	0.0	0.0

Staphylococcaceae	6.7	9.1	14.3	0.0	0.0	0.0
Veillonellaceae	6.7	0.0	0.0	0.0	0.0	0.0
Xanthomonadaceae	6.7	9.1	0.0	0.0	0.0	0.0
Cell-Shape	**70.0**	**75.0**	**80.0**	**85.0**	**90.0**	**95.0**
Coccus_shaped	26.7	18.2	28.6	0.0	0.0	0.0
Rod_shaped	60.0	72.7	71.4	100.0	100.0	100.0
Spiral_shaped	6.7	9.1	0.0	0.0	0.0	0.0
Unknown	6.7	0.0	0.0	0.0	0.0	0.0
Gram-Nature	**70.0**	**75.0**	**80.0**	**85.0**	**90.0**	**95.0**
Gram_Negative	40.0	36.4	28.6	0.0	0.0	0.0
Gram_Positive	60.0	63.6	71.4	100.0	100.0	100.0
Motility'	**70.0**	**75.0**	**80.0**	**85.0**	**90.0**	**95.0**
Motile	20.0	18.2	14.3	0.0	0.0	0.0
Nonmotile	53.3	54.5	57.1	50.0	66.7	50.0
Unknown	26.7	27.3	28.6	50.0	33.3	50.0
Oxygen-Requirement	**70.0**	**75.0**	**80.0**	**85.0**	**90.0**	**95.0**
Facultative_Anerobe	13.3	18.2	28.6	25.0	0.0	0.0
Microaerophilic	6.7	9.1	0.0	0.0	0.0	0.0
Obligate_Aerobe	60.0	63.6	57.1	50.0	66.7	100.0
Obligate_Anerobe	13.3	9.1	14.3	25.0	33.3	0.0
Unknown	6.7	0.0	0.0	0.0	0.0	0.0
Sporulation	**70.0**	**75.0**	**80.0**	**85.0**	**90.0**	**95.0**
Nonsporulating	60.0	54.5	71.4	50.0	66.7	50.0
Unknown	40.0	45.5	28.6	50.0	33.3	50.0

Table S4b: Comparison of various Phylogenetic and Phenotypic properties of top degree nodes of the microbial community interaction network for Lesional datasets of present study.

Phenotypes	**Degree Threshold**					
Phylum	**70.0**	**75.0**	**80.0**	**85.0**	**90.0**	**95.0**
Actinobacteria	62.5	62.5	40.0	0.0	0.0	0.0
Firmicutes	25.0	25.0	40.0	100.0	100.0	100.0
Proteobacteria	12.5	12.5	20.0	0.0	0.0	0.0
Class	**70.0**	**75.0**	**80.0**	**85.0**	**90.0**	**95.0**
Actinobacteria	62.5	62.5	40.0	0.0	0.0	0.0
Bacilli	25.0	25.0	40.0	100.0	100.0	100.0
Gammaproteobacteria	12.5	12.5	20.0	0.0	0.0	0.0
Order	**70.0**	**75.0**	**80.0**	**85.0**	**90.0**	**95.0**
Acidimicrobiales	12.5	12.5	20.0	0.0	0.0	0.0
Actinomycetales	50.0	50.0	20.0	0.0	0.0	0.0
Bacillales	25.0	25.0	40.0	100.0	100.0	100.0
Xanthomonadales	12.5	12.5	20.0	0.0	0.0	0.0
Family	**70.0**	**75.0**	**80.0**	**85.0**	**90.0**	**95.0**
Actinomycetaceae	12.5	12.5	0.0	0.0	0.0	0.0
Bacillaceae_2	12.5	12.5	20.0	0.0	0.0	0.0
Dermacoccaceae	12.5	12.5	0.0	0.0	0.0	0.0
Iamiaceae	12.5	12.5	20.0	0.0	0.0	0.0
Intrasporangiaceae	12.5	12.5	20.0	0.0	0.0	0.0
Micrococcaceae	12.5	12.5	0.0	0.0	0.0	0.0
Staphylococcaceae	12.5	12.5	20.0	100.0	100.0	100.0
Xanthomonadaceae	12.5	12.5	20.0	0.0	0.0	0.0
Cell-Shape	**70.0**	**75.0**	**80.0**	**85.0**	**90.0**	**95.0**
Coccus_shaped	37.5	37.5	40.0	100.0	100.0	100.0
Rod_shaped	37.5	37.5	40.0	0.0	0.0	0.0
Sphere_shaped	25.0	25.0	20.0	0.0	0.0	0.0
Gram-Nature	**70.0**	**75.0**	**80.0**	**85.0**	**90.0**	**95.0**
Gram_Negative	25.0	25.0	40.0	100.0	100.0	100.0
Gram_Positive	75.0	75.0	60.0	0.0	0.0	0.0
Motility'	**70.0**	**75.0**	**80.0**	**85.0**	**90.0**	**95.0**
Motile	25.0	25.0	20.0	0.0	0.0	0.0
Nonmotile	50.0	50.0	40.0	100.0	100.0	100.0
Unknown	25.0	25.0	40.0	0.0	0.0	0.0

Oxygen-Requirement	**70.0**	**75.0**	**80.0**	**85.0**	**90.0**	**95.0**
Facultative_Anerobe	12.5	12.5	20.0	100.0	100.0	100.0
Obligate_Aerobe	75.0	75.0	80.0	0.0	0.0	0.0
Obligate_Anerobe	12.5	12.5	0.0	0.0	0.0	0.0
Sporulation	**70.0**	**75.0**	**80.0**	**85.0**	**90.0**	**95.0**
Nonsporulating	62.5	62.5	60.0	100.0	100.0	100.0
Unknown	37.5	37.5	40.0	0.0	0.0	0.0

Table S5: Subject information

PATIENT ID	GENDER/AGE	AGE	TYPE OF VITILIGO
C	Female	29 years	Non-segmental, Vulgaris
O	Female	23 years	Non-segmental, Vulgaris
A	Female	30 years	Non-segmental, Vulgaris
P	Female	42 years	Non-segmental, Vulgaris
B	Female	28 years	Non-segmental, Vulgaris
Z	Female	35 years	Non-segmental, Vulgaris
N	Male	22 years	Non-segmental, Vulgaris
R	Male	25 years	Non-segmental, Vulgaris
Y	Male	24 years	Non-segmental, Vulgaris
X	Male	27 years	Non-segmental, Vulgaris

Table S6: MID sequences used for primer designing

Primer No.	MID sequence
27F1	AGACTCGACGT
27F2	AGTACGAGAGT
27F3	AGTACTACTAT
27F4	AGTAGACGTCT
27F5	AGTCGTACACT
27F6	GTGTACGACGT
27F7	ACACAGTGAGT
27F8	ACACTCATACTA
27F9	ACAGACAGCGT
27F10	ACAGACTATAT
27F11	ACAGAGACTCT
27F12	ACAGCTCGTGT
27F13	ACAGTGTCGAT
27F14	ACGAGCGCGCT
27F15	ACGATGAGTGT
27F16	ACGCGAGAGAT
27F17	ACGCTCTCTCT
27F18	ACGTCGCTGAT
27F19	ACGTCTAGCAT
27F20	ACTAGTGATAT

Table S7: Sample Ids, sampling sites and primers used for each sample in this study

Region A		
Primer	Sample ID	Site
27F1	Y1N1	Hand
27F2	Y1V1	
27F3	B1N2	Leg
27F4	B1V2	
27F5	B1N3	Leg
27F6	B1V3	
27F7	C1N1	Hand
27F8	C1V1	
27F9	Z1N2	Leg
27F10	Z1V2	
27F11	C1N3	Leg
27F12	C1V3	
27F13	U1N1	Leg
27F14	U1V1	
27F15	Y1N2	Leg
27F16	Y1V2	
27F17	U1N3	Hand
27F18	U1V3	
27F19	Z1N1	Leg
27F20	Z1V1	

Region B		
Primer	Sample ID	Site
27F1	A1N1	Leg
27F2	A1V1	
27F3	A1N3	Hand
27F4	A1V3	
27F5	P1N3	Hand
27F6	P1V3	
27F7	P1N4	Leg
27F8	P1V4	
27F9	R1N1	Leg
27F10	R1V1	
27F11	R1N2	Leg
27F12	R1V2	
27F13	V1N2	Hand
27F14	V1V2	
27F15	V1N4	Leg
27F16	V1V4	
27F17	X1N1	Hand
27F18	X1V1	
27F19	X1N4	Leg
27F20	X1V4	

Table S8: A list of differentially abundant taxa (RDP classification) between lesional and non-lesional skin obtained using the Wilcoxon test coupled to a bootstrapping approach. In each iteration of the bootstrap method, taxa with significantly different abundance were initially identified using Benjamini-Hochberg p-value correction at an FDR of 0.0001. Subsequently, taxa which were observed as having a significantly different abundance (post BH correction) in at least 99.5% of iterations were retained and are shown below. Samples in this analysis were obtained from anatomically similar sites.

Taxonomic Level		***Phylum Affiliation***	***Relative Contribution (%)***		***Median, Range (%)***	
			Normal	***Vitiligo***	***Normal***	***Vitiligo***
**GENUS**	Dermacoccus	Actinobacteria	0.050	0.240	0.006, 0.000-1.047▲	0.000, 0.000-0.473
	Brevibacillus	Firmicutes	0.010	0.040	0.006, 0.000-0.261▲	0.000, 0.000-0.044
	Roseomonas	Proteobacteria	0.040	0.070	0.050, 0.006-0.167▲	0.017, 0.000-0.234
	Novosphingobium	Proteobacteria	0.020	0.050	0.050, 0.000-0.099▲	0.013, 0.000-0.075
	Proteiniclasticum	Firmicutes	0.020	0.010	0.010, 0.000-0.061▲	0.008, 0.000-0.111
	Pelomonas	Proteobacteria	0.020	0.120	0.006, 0.000-0.467▲	0.000, 0.000-0.067
	Achromobacter	Proteobacteria	0.010	0.020	0.000, 0.000-0.073▲	0.000, 0.000-0.043
	Cellvibrio	Proteobacteria	0.000	0.030	0.012, 0.000-0.220▲	0.000, 0.000-0.013
	Geminicoccus	Proteobacteria	0.010	0.020	0.005, 0.000-0.348▲	0.000, 0.000-0.037
	Nocardia	Actinobacteria	0.000	0.020	0.005, 0.000-0.200▲	0.000, 0.000-0.078
	Leucobacter	Actinobacteria	0.010	0.010	0.000, 0.000-0.050	0.007, 0.000-0.035▲
	Yimella	Actinobacteria	0.090	0.020	0.000, 0.000-0.334	0.014, 0.000-0.545▲
	Aeromicrobium	Actinobacteria	0.030	0.040	0.012, 0.000-0.132	0.025, 0.000-0.098▲
	Varibaculum	Actinobacteria	0.010	0.020	0.000, 0.000-0.063	0.007, 0.000-0.156▲
	Skermanella	Proteobacteria	0.150	0.060	0.011, 0.000-0.295	0.059, 0.000-0.675▲
	Erythrobacter	Proteobacteria	0.010	0.010	0.000, 0.000-0.030	0.007, 0.000-0.044▲
	Vibrio	Proteobacteria	0.010	0.030	0.000, 0.000-0.201	0.007, 0.000-0.104▲
	Atopostipes	Firmicutes	0.010	0.020	0.000, 0.000-0.098	0.007, 0.000-0.085▲
	Leuconostoc	Firmicutes	0.010	0.010	0.000, 0.000-0.074	0.007, 0.000-0.044▲
	Erythromicrobium	Proteobacteria	0.010	0.020	0.000, 0.000-0.332	0.005, 0.000-0.062▲

**FAMILY**	Flavobacteriaceae	Bacteroidetes	1.080	1.300	1.178, 0.114-3.050▲	0.658, 0.235-4.255
	Beijerinckiaceae	Proteobacteria	0.070	0.100	0.021, 0.000-0.322▲	0.006, 0.000-0.294
	Enterococcaceae	Firmicutes	0.100	0.150	0.046, 0.000-0.272▲	0.020, 0.000-0.385
	Rhodospirillaceae	Proteobacteria	0.150	0.070	0.021, 0.000-0.269	0.057, 0.016-0.532▲
	Vibrionaceae	Proteobacteria	0.010	0.030	0.005, 0.000-0.220	0.006, 0.000-0.100▲
	Eubacteriaceae	Firmicutes	0.010	0.010	0.000, 0.000-0.053	0.004, 0.000-0.024▲
	Promicromonosporaceae	Actinobacteria	0.010	0.010	0.000, 0.000-0.058	0.003, 0.000-0.102▲
	Micromonosporaceae	Actinobacteria	0.020	0.090	0.000, 0.000-0.326	0.012, 0.000-0.063▲
**ORDER**	Alphaproteobacteria_incertae_sedis	Proteobacteria	0.000	0.020	0.005, 0.000-0.309▲	0.000, 0.000-0.035
	Vibrionales	Proteobacteria	0.010	0.030	0.005, 0.000-0.212	0.006, 0.000-0.097▲
	Myxococcales	Proteobacteria	0.020	0.000	0.000, 0.000-0.031	0.011, 0.000-0.057▲
**CLASS**	Acidobacteria_Gp1	Acidobacteria	0.01	0.01	0.000, 0.000-0.028	0.007, 0.000-0.040▲

Table S9: A list of differentially abundant OTUs between lesional (Vitiligo) and non-lesional (Normal) skin obtained using the Wilcoxon test coupled to a bootstrapping approach. In each iteration of the bootstrap method, taxa with significantly different abundance were initially identified using Benjamini-Hochberg p-value correction at an FDR of 0.0001. Subsequently, taxa which were observed as having a significantly different abundance (post BH correction) in at least 99.5% of iterations were retained and are shown below. Samples in this analysis were obtained from anatomically similar sites.

OTU	***Phylum Affiliation***	***Relative Contribution (%)***		***Median, Range (%)***	
		Normal	***Vitiligo***	***Normal***	***Vitiligo***
OTU_105_Skermanella	Proteobacteria	0.13	0.05	0.051, 0.000-0.467 ▲	0.010, 0.000-0.240
OTU_208_Bacillus	Firmicutes	0.01	0.01	0.007, 0.000-0.090 ▲	0.000, 0.000-0.225
OTU_593_Aeromicrobium	Actinobacteria	0.02	0.02	0.005, 0.000-0.071 ▲	0.000, 0.000-0.094
OTU_606_Hahella	Proteobacteria	0.00	0.02	0.005, 0.000-0.018 ▲	0.000, 0.000-0.084
OTU_946_Clostridiaceae	Firmicutes	0.02	0.01	0.012, 0.000-0.047 ▲	0.000, 0.000-0.066
OTU_1078_Massilia	Proteobacteria	0.02	0.02	0.018, 0.000-0.071 ▲	0.009, 0.000-0.045
OTU_1111_Planococcaceae	Firmicutes	0.01	0.00	0.005, 0.000-0.079 ▲	0.000, 0.000-0.009
OTU_1621_Anaerococcus	Firmicutes	0.01	0.01	0.008, 0.000-0.087 ▲	0.000, 0.000-0.080
OTU_1674_Schlegelella	Proteobacteria	0.01	0.01	0.015, 0.000-0.054 ▲	0.000, 0.000-0.040
OTU_1763_Sanguibacter	Actinobacteria	0.03	0.03	0.019, 0.000-0.126 ▲	0.008, 0.000-0.275
OTU_1_Enterobacteriaceae	Proteobacteria	0.99	0.70	0.104, 0.000-4.800	0.221, 0.000-1.830 ▲
OTU_19_Xanthomonadaceae	Proteobacteria	0.28	0.26	0.077, 0.029-0.976	0.132, 0.000-0.616 ▲
OTU_32_Propionibacterium	Actinobacteria	7.73	12.19	1.312, 0.217-48.211	2.862, 0.188-51.543 ▲
OTU_42_Nocardioides	Actinobacteria	0.16	0.23	0.153, 0.059-0.261	0.176, 0.030-0.652 ▲
OTU_50_Brevundimonas	Proteobacteria	0.42	0.60	0.162, 0.071-1.450	0.317, 0.000-1.616 ▲
OTU_85_Roseomonas	Proteobacteria	0.01	0.03	0.005, 0.000-0.050	0.025, 0.000-0.070 ▲
OTU_115_Bacilli	Firmicutes	0.35	0.45	0.043, 0.000-1.582	0.112, 0.000-1.175 ▲
OTU_143_Saccharopolyspora	Actinobacteria	0.02	0.10	0.004, 0.000-0.081	0.025, 0.000-0.355 ▲
OTU_566_Tessaracoccus	Actinobacteria	0.11	0.14	0.084, 0.020-0.395	0.109, 0.000-0.314 ▲
OTU_651_Micropruina	Actinobacteria	0.01	0.01	0.000, 0.000-0.035	0.008, 0.000-0.040 ▲
OTU_859_Thermomonas	Proteobacteria	0.07	0.10	0.043, 0.010-0.304	0.070, 0.000-0.342 ▲
OTU_1577_Sphingomonas	Proteobacteria	0.13	0.36	0.054, 0.006-0.336	0.102, 0.010-0.826 ▲
OTU_1938_Leifsonia	Actinobacteria	0.00	0.01	0.000, 0.000-0.025	0.005, 0.000-0.056 ▲
